# Investigating the Molybdenum Nitrogenase Mechanistic Cycle Using Spectroelectrochemistry

**DOI:** 10.1021/jacs.4c16047

**Published:** 2025-01-02

**Authors:** Kushal Sengupta, Justin P. Joyce, Laure Decamps, Liqun Kang, Ragnar Bjornsson, Olaf Rüdiger, Serena DeBeer

**Affiliations:** Department of Inorganic Spectroscopy, Max Planck Institute for Chemical Energy Conversion, Mülheim an der Ruhr, Germany, 45470

## Abstract

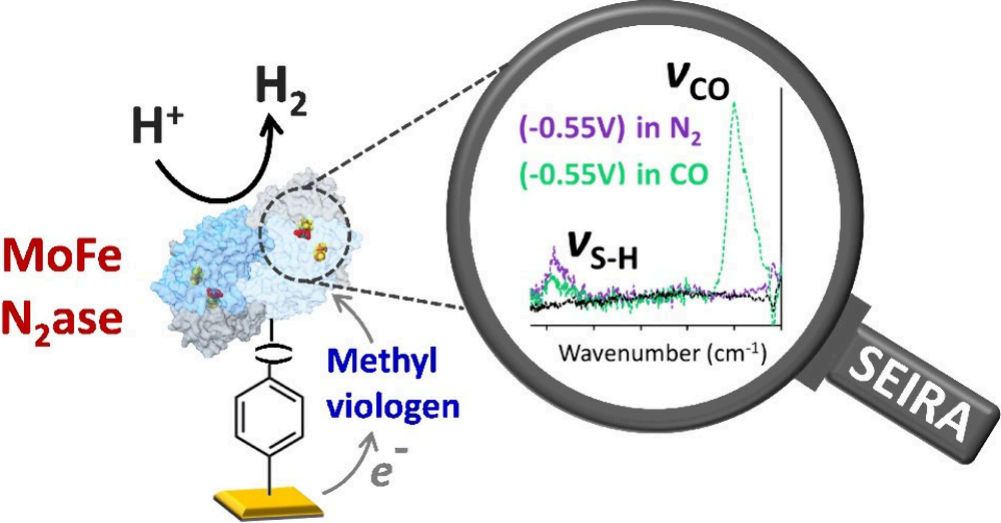

Molybdenum nitrogenase plays a crucial role in the biological nitrogen cycle by catalyzing the reduction of dinitrogen (N_2_) to ammonia (NH_3_) under ambient conditions. However, the underlying mechanisms of nitrogenase catalysis, including electron and proton transfer dynamics, remain only partially understood. In this study, we covalently attached molybdenum nitrogenase (MoFe) to gold electrodes and utilized surface-enhanced infrared absorption spectroscopy (SEIRA) coupled with electrochemistry techniques to investigate its catalytic mechanism. Our biohybrid system enabled electron transfer via a mild mediator, likely mimicking the natural electron flow through the P-cluster to FeMoco, the enzyme’s active site. For the first time, we experimentally observed both terminal and bridging S–H stretching frequencies, resulting from the protonation of bridging sulfides in FeMoco during turnover conditions providing direct evidence of their role in catalysis. These experimental observations are further supported by QM/MM calculations. Additionally, we investigated CO inhibition, demonstrating both CO binding and unbinding dynamics under electrochemical conditions. These insights not only advance our understanding of the mechanistic cycle of molybdenum nitrogenase but also establish a foundation for studying alternative nitrogenases, including vanadium and iron nitrogenases.

## 1 Introduction

**Figure 1:**
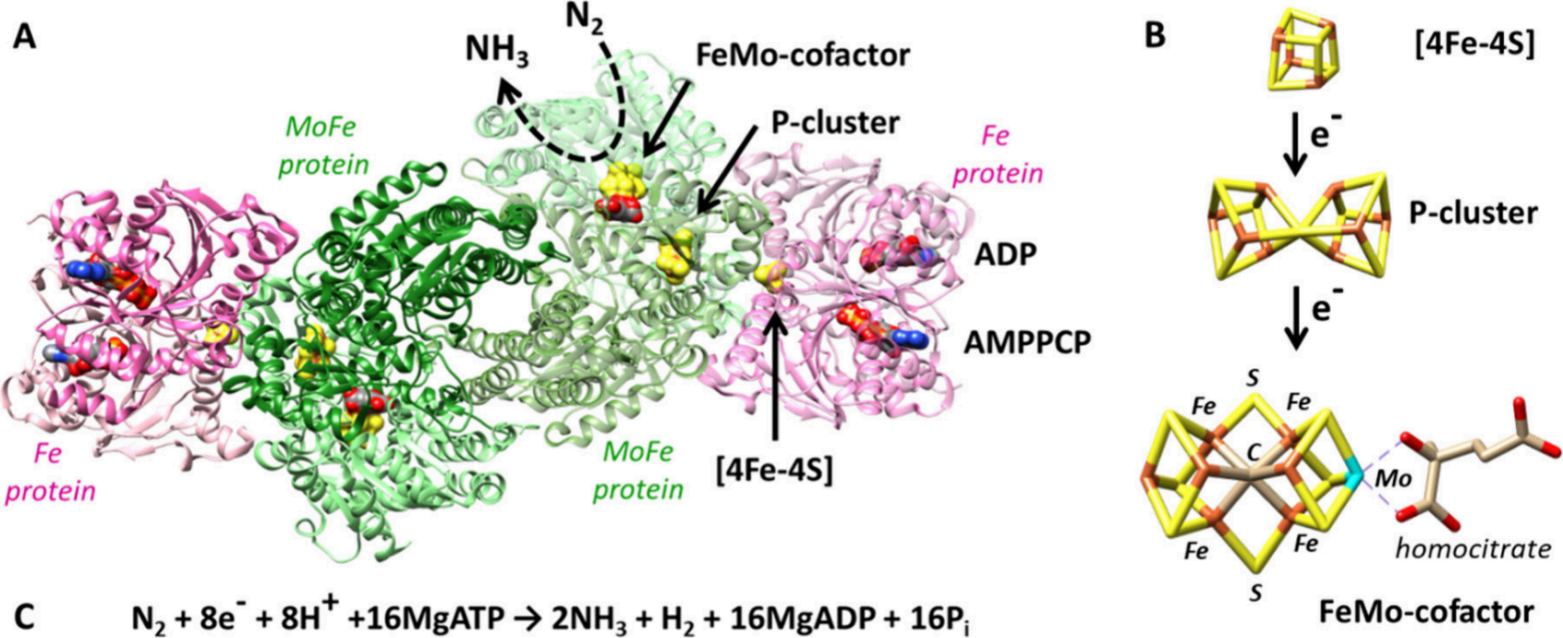
(A) Crystal structure of Mo-dependent N_2_ase from *Azotobacter vinelandii*, showing the Fe:MoFe complex in the presence of ATP analogs (PDB: 4WZA). (B) Stick models of the [4Fe-4S] cluster, P-cluster, and the FeMo-cofactor, showing the electron transfer direction. (C) The limiting stoichiometry of N_2_ reduction to NH_3_ by Mo N_2_ase.

Nitrogen (N), an essential element for life, constitutes 78% of Earth’s atmosphere in the form of chemically inert dinitrogen (N_2_). The conversion of N_2_ to ammonia (NH_3_), a bioavailable form of nitrogen, plays a pivotal role in the biogeochemical nitrogen cycle, with significant agronomic and economic implications.^1,2^ Additionally, NH_3_ also has potential as an alternative fuel and a hydrogen storage medium.^3,4^ Ammonia is primarily produced through two processes: biological N_2_ fixation (BNF) in diazotrophic microbes (∼32%), and the industrial Haber–Bosch process (HBP, ∼65%), with the remaining 3% resulting from lightning.^5−8^ Although efficient, the HBP is energy-intensive, requiring high temperatures (∼500 °C) and pressures exceeding 20 MPa. The process consumes 1–2% of the world’s annual energy production and contributes approximately 1.6% of global CO_2_ emissions. In contrast, BNF occurs at ambient temperature and pressure and is primarily limited to a group of bacteria and archaea known as diazotrophs,^5,9,10^ and very recently identified in a marine alga,^11^ which perform this function through a complex set of metalloenzymes called nitrogenases (N_2_ases). N_2_ases are categorized into three subclasses based on their cofactors: Mo, V, and Fe.^6,12^ Each N_2_ase is a two-component protein system, comprising a [4Fe-4S] cluster-containing iron protein (FeP) acting as a reductase, and a catalytic protein (MFe, where M = Mo, V, or Fe) (Figure 1A). The catalytic protein harbors two metal cofactors: the [8Fe-7S] P-cluster, involved in electron transfer, and the carbide-containing [7Fe-M-9S-C] FeMco cluster, the active site for catalysis (Figure 1B).^6,8,13−16^ The Mo-dependent N_2_ase from *Azotobacter vinelandii* is the most extensively studied and also the focus of this work.

**Figure 2:**
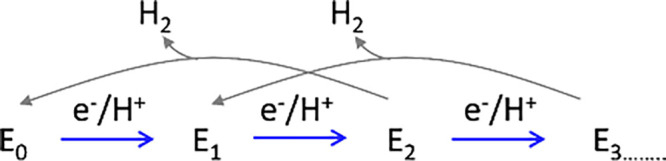
A simpler version of the early steps of the Lowe–Thorneley cycle emphasizing the proposed states formed upon transfer of 1e^–^/1H^+^ to the active site and the subsequent release of H_2_ leading to “off-pathway” states.

Mo-N_2_ase is considered the most efficient isozyme, requiring 8 electrons (e^–^) and 8 protons (H^+^) to produce 2 mol of NH_3_ from 1 mol of N_2_ (Figure 1C), along with 1 mol of H_2_. This process necessitates the hydrolysis of 16 adenosine triphosphate (MgATP) molecules.^13,17^ The FeP reductase, in addition to its [4Fe-4S] cluster, binds ATP (or similar nucleoside phosphate) to each monomer. This is believed to facilitate its interaction with MoFe, inducing conformational changes and electron transfer (ET) from the P-cluster to the FeMoco, the catalytic site for substrate binding and reduction. This electron deficit is then replenished by ET from FeP (Figure 1B).^8,18−21^ This cycle, known as the Fe protein cycle, alternates with the MoFe cycle, resulting in substrate activation and reduction. Each FeP cycle delivers 1e^–^ and 1H^+^ to the active site, through the association and dissociation of the two protein components, hydrolysis of 2 molecules of MgATP, and release of inorganic phosphate (P_i_), which repeats 8 times to achieve the stoichiometric reduction of one molecule of N_2_.^13,22^ The MoFe protein cycle operates according to the Lowe-Thorneley (LT) kinetic model, as depicted in a simplified partial form in Figure 2.^3,6,13,23^ In this model, e^–^ and H^+^ are accumulated within the cofactor. The resting state of the enzyme is represented as E_0_, while subsequent states, such as E_1_, E_2_, and so forth (Figure 2) denote configurations that have accumulated one or more e^–^/H^+^. It is hypothesized that these e^–^/H^+^ may be stored by protonation of the bridging sulfides and/or as hydrides bound to the iron centers depending on the specific E_n_ state.^13,24^ Following the LT model, it is generally accepted that, under slow transfer of electrons or protons for further reduction, or in the absence of substrate, the MoFe intermediates release the stored H^+^/H^–^ as H_2_, thereby reverting to an earlier intermediate state (Figure 2).^7,8,17^

It is noteworthy that advanced spectroscopic techniques have provided key insights into our current understanding of nitrogenase^14,25−29^ and have been particularly important for characterizing the nature of protonated intermediates.^30−32^ Utilizing a combination of EXAFS and QM/MM calculations, our research group has argued that the first protonation on FeMoco occurs at a bridging sulfide,^26^ while an alternative interpretation has been argued based on EPR studies of iron-only nitrogenase.^33^ Arguably, in both cases the experimental evidence is indirect. Stronger evidence for the presence of protonated sulfides comes from ^2^H Mims ENDOR on D_2_O turnover samples, which implicates the presence of terminal or bridging SH group in the E_4_(4H) state of nitrogenase.^32^ While the authors indicate that the nature of the SH group, as either bridging or terminal, cannot be assigned, they note that the sulfur must still be bound to the cluster, in contrast to protein crystallographic studies, which have suggested complete loss of the S2B sulfur during turnover.^34,35^ Theoretical studies have suggested that protonated bridging sulfides can change from a bridging to terminal configuration in reduced states E_N_ > 1.^36−38^ Interestingly to date, there have been no experimentally reported S–H stretching frequencies for any nitrogenase FeMco cluster, though such data would be highly desirable in order to experimentally evaluate mechanistic proposals. Much of the research on nitrogenase has focused on elucidating the mechanism by which it operates to achieve the reduction of its physiological substrates, including nitrogen, and protons. It has been now established that all nitrogenase variants can also reduce a variety of other substrates (including acetylene, carbon dioxide, diazene, azides, hydrazine, etc.) with varying degrees of both efficiency and product distributions.^7^ Numerous research groups have contributed to advancing our understanding of the nitrogenase mechanism by investigating the enzyme’s reactivity with these alternative substrates providing valuable insights into potential applications of nitrogenase for carrying out useful reductions beyond nitrogen fixation.^7,17,39−44^ A particular alternative substrate/inhibitor that has garnered significant interest over the years is carbon monoxide (CO).^7,34,45−48^ Although CO is an inhibitor of nitrogen reduction in Mo-N_2_ase, it does not affect proton reduction.^46^ Furthermore, CO serves as a substrate for alternative nitrogenases, such as V-N_2_ase.^7,49^ Significant progress has been made over the past few decades, especially in recent years, in understanding the nitrogenase mechanism through experimental approaches such as spectroscopy, relevant biochemical analyses, and theoretical calculations.^5,6,8,12,13,45,50,51^ However, although our current understanding has vastly improved, there remain numerous open questions and poorly understood steps in the mechanism. One of the experimental limitations is the difficulty in isolating and characterizing relevant intermediates for subsequent studies, as N_2_ reduction relies on the dynamic interaction between the two proteins, ATP hydrolysis, the use of ATP-regenerating systems, and the ability to quench the reaction at the desired time point.^52^

Given these challenges, there has been considerable interest in exploring the MoFe catalytic protein, independent of the reductase protein and ATP hydrolysis. In recent years, it has been demonstrated that both photocatalytically and electrocatalytically driven systems can be utilized in place of the FeP in order to drive N_2_ reduction.^52−56^ In the case of photocatalytic systems utilizing CdS quantum dots, these studies have also been coupled to spectroscopy in order to reveal the nature of key intermediates in N_2_ reduction.^57,58^ In contrast, the utilization of spectroscopy in combination with nitrogenase biohybrid electrocatalysts has been far less explored. A notable exception to this is the recent work from Vincent and co-workers in which MoFe protein was either immobilized via covalent attachment to carbon particles,^56^ or stabilized with Nafion and trapped between a carbon paper and the ATR prism.^59^ In both of these studies, a series of mediators were used for electron transfer. Interestingly, they were able to observe CO binding in the MoFe, however, unlike native MoFe protein, CO binding to the enzyme in such conditions inhibited proton reduction.^56,59^ This suggests that the observed intermediates may be different than what is found in the native enzyme.

An important open question for both the photo- and electrocatalytic MoFe systems is whether electron transfer occurs to the P-cluster, to FeMoco, or potentially to both cofactors.^60^ Ideally for the spectroscopic intermediates to mimic the native system, one would prefer that a biomimetic pathway via the P-cluster is followed.^8,61^ In the case of both the photo- and electrocatalytic hybrids, the use of strong reductants (CdS nanorods (*E*^0^ > −0.8. V vs NHE) in the photocatalysts and either cobaltocene (*E*^0^ ∼ −0.96 V vs NHE) or Eu(II) chelates (*E*^0^ > −1.1 V vs NHE) in the electrocatalytic systems), means that electron transfer directly to FeMoco *could become possible*, potentially resulting in processes that are off path relative to the native system. Dukovic and co-workers previously discussed the possibility of electron transfer via the P-cluster or directly to FeMoco.^62^

In the present study, we take an alternative approach to previous nitrogenase biohybrid studies and instead utilize a mild mediator (methyl viologen *E*^0∼^, −0.47 V), with a redox potential between the reported potentials of the P-cluster (P^N^/P^1+^, −0.23 V)^8,55^ and FeMoco (E^0^/E^n^, −0.59 V).^55^ We note that we have considered the values −0. 59 V for FeMoco, as to our knowledge that is the only value that could be confirmed to represent an E^0^/E^n^ couple. Given these limiting assumptions, utilizing methyl viologen, the approach reported herein, should increase the possibility that electron transfer from the electrode/mediator to the cofactor occurs via the P-cluster, aligning with our aim to emulate the wild-type (WT) protein’s behavior. Arguably, upon covalent attachment, the redox potentials of the two clusters within the protein may deviate from the reported values due to conformational changes, creating a possibility for ET from the mediator to FeMoco. However, measurement of these redox potentials in our modified electrode system is, at this point, not feasible which makes it challenging to conclusively assign the ET pathway. Nonetheless, the choice of mediator was informed by existing knowledge to align with the intended electron transfer pathway. Additionally, we note that such mild mediators were likely not explored in the electrochemical or spectroelectrochemical studies of MoFe, discussed above, due to the fact that mediators like methyl viologen are not competent to reduce MoFe in solution or when it is adsorbed on an electrode, in the absence of FeP.^54,63,64^ An exception to this is a report by Minteer and co-workers, who immobilized His-tagged MoFe on an electrode via coordinate covalent interaction between nickel and the His-tag on the protein. This assembly exhibited the reduction of azides in the presence of viologen mediators, which was observed through cyclic voltammetry experiments.^65^ Herein, we show that a catalytically active MoFe biohybrid can be obtained with a mild reductant. These findings might suggest that similar to the conformationally induced gating of MoFe by FeP, which enables electron transfer in the native system, our approach of immobilization of MoFe via direct covalent attachment (in contrast to simply adsorption or coordinate interactions on the electrode surface) have made the ET to FeMoco facile. By coupling our steady state turnover electrochemical studies of our biohybrid system to surface-enhanced infrared absorption (SEIRA), we are able to provide ***the first direct experimental evidence for the presence of both bridging and terminal S–H stretching frequencies in MoFe during turnover conditions*** in the presence of N_2_, as well as in the presence of CO, thus providing clear experimental support for the role of the bridging sulfurs in the enzymatic reaction mechanism. We have also gained insight into CO unbinding and characterized the nature of CO bound to a range of E_n_ intermediates. The relevance of the observed intermediates to the mechanism of the native protein is highlighted.

## 2 Experimental Details

### 2.1 Materials and Protein Purification

All reagents, including chemicals and buffers, were obtained from Sigma-Aldrich or Fisher Scientific and were used without further purification. Ultrapure water was obtained from a Millipore unit (18 MΩ cm) and used to prepare all aqueous solutions. Argon, dinitrogen, CO_2_, and CO gases were purchased from Westfalen and passed through an activated copper catalyst to remove any traces of dioxygen before use. ^13^CO gas was purchased from EURIO-TOP GmbH.

WT MoFe (^WT^MoFe) and ^Apo^MoFe proteins were purified from *Azotobacter vinelandii wt* and DJ2115 (*genotype: nifD*^*strep*^*, ΔnifB:kanR*) strains, respectively, following published procedures.^66,67^ Both WT and ^Apo^MoFe proteins were eluted in an aqueous buffer with 20 mM TRIS pH 7.4, and 200 mM NaCl. Notably, the use of dithionite was avoided during the purification process, as dithionite and its oxidized products have been shown in the literature to potentially affect nitrogenase chemistry, particularly in electrochemical studies.^68^ The purities of these proteins were >95% based on SDS-PAGE analysis with Coomassie staining. All manipulation of proteins and buffers was performed under an N_2_ atmosphere. For electrochemical and spectroelectrochemical experiments in deuterium buffers, aliquots of the purified proteins were further exchanged into the corresponding storage pD 7 buffers to ensure the exchange of protons to deuterium.

### 2.2 Preparation of MoFe Nitrogenase Modified Electrodes (MoFe_Au_)

**Figure 3:**
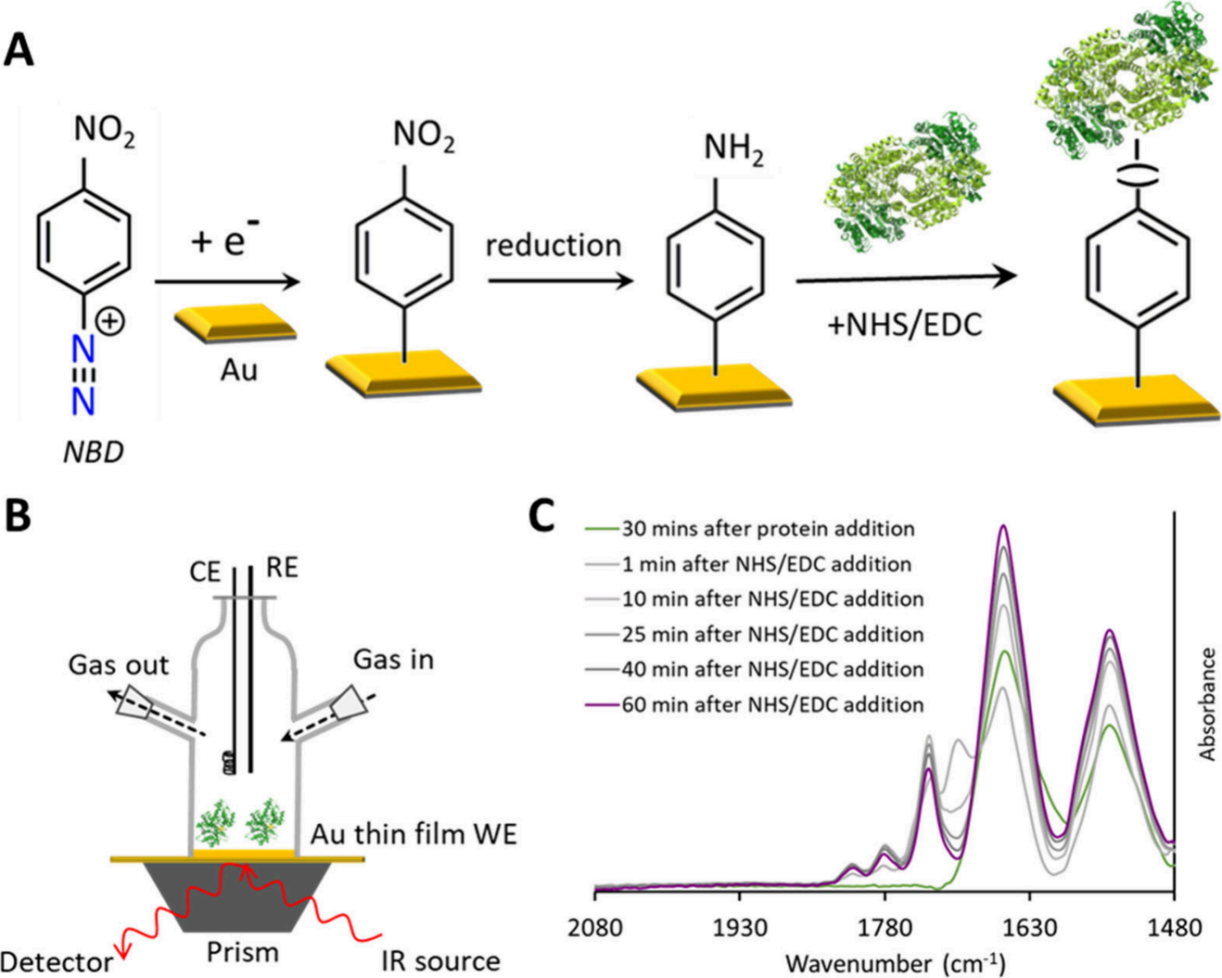
Schematic representation of the immobilization strategy of MoFe protein on modified Au electrodes (A) and the in-house SEIRA-SEC cell setup (B). (C) SEIRA spectra of the MoFe protein upon addition to the modified electrode and after addition of NHS/EDC.

A gold rotating disk electrode (RDE) (5 mm disk OD, Pine Instruments) was cleaned using piranha solution (and if needed was polished using 0.05 μm Alumina MasterPrep Polishing Suspension (Buehler, Esslingen, Germany)). Subsequently, the electrode was sonicated in Milli-Q water for 5 min to remove any residual polishing medium. To ensure complete cleaning, the electrode was electrochemically cleaned in a spectroelectrochemical cell containing 0.5 M H_2_SO_4_ saturated with argon. This involved repeating potential scans between 0.2 and 1.8 V versus NHE for ten cycles.^69^ The electrode was then rinsed thoroughly with water and dried prior to further modification. This electrode then underwent surface modification using 4-nitrobenzenediazonium (NBD) salt. Chronoamperometry was performed on the clean electrode for 30 s at 0.1 V (vs NHE) in 1 mM solution of NBD in ACN, followed by cyclic voltammetry (2 scans, 0.2 V to −1.0 V vs NHE at 100 mV/s) in 0.1 M NaClO_4_ solution (1:9= ethanol: water) which would ensure the reduction of the nitro groups to amino groups, thus modifying the electrode to a monolayer of exposed -NH_2_ groups.^70^ Following surface modification, the electrode was transferred to an N_2_-filled glovebox (MBRAUN, Garching, Germany) to eliminate any adsorbed oxygen, where it was left undisturbed for approximately 15 min. A solution of 20 μM protein in 20 mM TRIS buffer with 50 mM NaCl (pH 7) was then applied to the electrode surface and allowed to incubate for 30 min. Subsequently, *N*-hydroxysuccinimide (NHS) and 1-Ethyl-3-(3-(dimethylamino)propyl)carbodiimide (EDC) were added to final concentrations of 2.8 mM and 5.6 mM, respectively, to covalently link the protein to the electrode surface. The reaction was allowed to proceed for 60–80 min. Finally, the electrode was rinsed thoroughly with the corresponding buffer to remove any unbound materials. Figure 3A provides a schematic illustration of the electrode modification and protein attachment steps.

### 2.3 Electrochemistry

The electrochemical measurements were conducted using a standard three-electrode cell, equipped with a saturated calomel reference electrode (SCE), which was isolated in a side arm filled with 0.1 M KCl solution. The stability of the reference electrode potential was periodically verified against (hydroxymethyl)ferrocene, calibrated at 0.42 V vs NHE. All potential control and data acquisition were facilitated by a multichannel potentiostat (Princeton Applied Research, Oak Ridge, USA). Unless otherwise specified, the electrochemical experiments were carried out under the following standardized conditions: ambient room temperature maintained between 22 and 25 °C; an electrolyte solution consisting of 100 mM MOPS buffer at pH 7, supplemented with 150 μM methyl viologen (MV) as the electron transfer mediator. The cell was maintained under an atmosphere of nitrogen (1 atm) or other specified gases. For gases other than N_2_, the buffer solutions (excluding MV) were purged with the designated gas (e.g., CO) for approximately 20–30 min prior to the measurements. Cyclic voltammetry experiments were conducted at a scan rate of 20 mV/s without electrode rotation, to evaluate the electrochemical behavior under static conditions. For bulk electrolysis (BE) experiments, a custom-designed, airtight electrochemical cell (5 mL volume, Figure S1) was utilized. The working electrode was maintained at a specified constant potential. A magnetic stir bar, rotating at 500 rpm, was employed to ensure consistent diffusion throughout the experiments. The buffer volume was measured to 2 mL, ensuring a 3 mL headspace gas in the reaction cell. Before commencing the BE experiments, the cell was thoroughly purged with the appropriate gas, such as N_2_ or CO, for 10 min. This step was crucial to remove any residual hydrogen or other potential contaminants that could influence the experimental outcomes.

### 2.4 Gas Chromatography

The headspace gas collected from the electrochemical cells was systematically analyzed using a gas chromatography (GC) instrument (Thermo Fischer Scientific, Trace 1310). Samples were taken at regular time intervals to monitor the product formation over the course of the experiment. A gastight Hamilton Samplelock syringe was employed to withdraw exactly 100 μL of headspace gas at each specified time point. To ensure the integrity of the gas samples, it was crucial to thoroughly flush the syringe with Ar multiple times to eliminate any residual air or contaminants before inserting the syringe into the electrochemical cell and withdrawing 100 μL. For the GC analysis, Ar was used as the carrier gas, and TCD as the detector. To estimate the actual amount of hydrogen gas produced, the area under the hydrogen peak from the GC chromatogram was interpreted based on a standard curve generated from known amounts of hydrogen. This standard curve was measured under identical parameters as the experimental conditions. Consequently, in the representation of the figures illustrating the amount of hydrogen, the total headspace of the cell for the actual experiments has been taken into consideration.

### 2.5 Spectroelectrochemistry: Surfaced Enhanced Infrared Spectroscopy (SEIRA) Coupled to Electrochemistry (SEIRA-SEC)

The spectroelectrochemistry experiment was conducted using a homemade electrochemical cell equipped with a silicon prism functionalized with a nanostructured Au film, where the silicon prism functioned as the attenuated total reflectance (ATR) element and the Au film served as the working electrode, additionally enabling enhanced IR absorption (Figure 3B). The Au nanostructured film on a silicon prism was prepared via electroless deposition.^71^ Initially, the silicon prism was polished using alumina slurries, followed by thorough rinsing with ethanol and water. Subsequently, the prism was immersed in a 40% NH_4_F solution for approximately 2 min to remove the oxide layer from the silicon surface and terminate it with hydrogen.^72^ The Au deposition was carried out at 65 °C in a water bath by applying a mixture of 240 μL of plating solution (0.015 M NaAuCl_4_, 0.15 M Na_2_SO_3_, 0.05 M Na_2_S_2_O_3_, 0.05 M NH_4_Cl) and 80 μL of 2% HF to the hydrogen-terminated silicon prism surface. After 60 s, the Au deposition reaction was terminated by rinsing the prism with water. *It is noteworthy that a fresh plating solution should be prepared every couple of months to ensure optimal results*. The modified prism was then fitted to the electrochemical cell. Subsequently, the gold film was cleaned electrochemically, followed by functionalization with NBD, as previously detailed (Section 2.2). The cell was then transferred to a COY box filled with nitrogen, which housed a Bruker Tensor 27 Infrared Spectrometer equipped with a liquid N_2_ cooled MCT detector, and was left undisturbed overnight (∼12 h) to remove any adsorbed oxygen.

Before immobilization of the protein, a reference spectrum was recorded by averaging 500 scans in the corresponding experimental buffer solution. After recording the reference spectrum, the buffer solution was replaced with a 60 μM protein solution in the respective buffer, and FTIR spectra were recorded during this process (about 30 min). This allowed us to monitor the increase in the amide bands (∼1650 and ∼1550 cm^–1^). Following this process, NHS and EDC were added to the solution for the covalent attachment of the protein onto the modified electrode surface, using the same procedure and twice the concentrations discussed in Section 2.2. Throughout this period, FTIR measurements were employed to monitor the covalent attachment process. The initial increase in the amide bands, followed by their subsequent stabilization over time, provided insights into the duration (∼60 min) required for the attachment (Figure 3C, Figure S2). *Notably*, *in addition to the amide bands of the proteins, the stronger asymmetric and weaker symmetric carbonyl stretches (1745–1810 cm*^*–1*^*) of the NHS ester, formed during this process, could also be observed in our FTIR spectrum*.^73^ Afterward, the cell was rinsed a couple of times with the corresponding experimental buffer. Subsequently, 3 mL of the corresponding buffer was added to the cell, and the SEIRA-SEC measurements were initiated. For all these measurements, FTIR spectra were collected and averaged over 100 scans (equivalent to 80 s) for each condition while applying a fixed potential in the parallel bulk electrolysis. For the SEIRA measurements, the spectra were collected with a resolution of 2 cm^–1^, and the spectrometer aperture was set to 2.5 mm.

### 2.6 Attenuated Total Reflectance Fourier-Transform Infrared Spectroscopy (ATR-FTIR)

The ATR measurements were conducted using the same Bruker Tensor 27 Infrared Spectrometer, as the SEIRA measurements, housed within a COY box filled with nitrogen and equipped with a liquid nitrogen cooled MCT detector. For the measurements, 10 to 15 μL of the ∼100 μM protein solution was added to the ATR element (Bruker BIO-ATR II), and dried with a flow of argon gas for approximately 10 min. For subsequent reactions with follow-up conditions (like dithionite (DT) or MV), a solution of the respective substance was added onto the same window each time, allowing it to react for 10 min before drying over the flow of Ar gas (or any other gas mentioned in the experiment) and proceeding for data collection. The spectra were collected with a resolution of 2 cm^–1^ and were averaged over three scans.

### 2.7 Quantum Mechanical/Molecular Mechanical (QM/MM) Calculations

The MM model utilized in this study was previously reported^74^ and is based on the crystal structure of the MoFe protein in its E_0_ state (PDB ID: 3U7Q).^75^ The QM/MM calculations were performed using the ASH program,^76^ which interfaces the OpenMM library^77^ and the ORCA electronic structure software package.^78^ The MM region was described using the CHARMM36 force field,^79^ as used in previous QM/MM studies of FeMoco in various E_n_, and substrate-bound states.^36−38,74,80^ Geometry optimizations were performed using the geomeTRIC optimization library^81^ using HDLC internal coordinates.^82^ The covalent QM-MM boundaries were treated using a link atom-strategy and a charge-shifting scheme^83^ as implemented in ASH. A single QM region was employed, which includes FeMoco and its primary (homocitrate, Cys275, His442) and secondary coordination sphere (Val70, Arg96, Gln191, Ser192, His19, Arg359, Glu380, and Phe381). The r^2^ SCAN density functional was used to describe the QM region together with the D4 dispersion developed by Grimme and parametrized by Brandenburg.^84−86^ The calculations used the accurate integration grid in ORCA (“defgrid2”). The ZORA scalar relativistic Hamiltonian was used with the ZORA-def2-TZVP basis set on all Fe and S centers and the interstitial carbide. The same ZORA-def2-TZVP basis set was used for the substrates H and CO. The all-electron SARC-ZORA-TZVP basis set was used for Mo. The remaining atoms were treated with the ZORA-def2-SVP basis set. The Coulomb integrals were treated with the Split RI-J approximation together with a decontracted auxiliary basis set (“SARC/J”). The vibrational frequencies of selected functional groups were calculated by diagonalizing the partial QM/MM Hessian matrix of the ligand coordinates and binding iron centers. The calculated vibrational frequencies are not scaled.

### 2.8 Multicomponent Fitting of the ^WT^MoFe_Au_ Bound CO Vibration

The SEIRA spectra acquired under pH 7 and pD 7 conditions in the presence of CO were quantitatively analyzed using the LMFIT Python library.^87^ The original experimental spectra, recorded at various potentials, were initially truncated to the region of interest (2100 to 1750 cm^–1^) and were subtracted from the initial spectra recorded at OCP. Each spectrum’s offset was then adjusted to correct the baseline, using the region between 2100 and 2050 cm^–1^ as a reference, before being imported for peak fitting. Peak fitting was performed globally, meaning all spectra were fit simultaneously with shared parameters, and the minimization objective was the combined residual of all spectra. The fitting model was constructed with various Gaussian peaks, each representing a specific species. A typical Gaussian function is in this form, with A, μ, and σ representing the amplitude, the mean (center) of the peak, and the standard deviation, which determines the width of the peak.
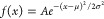


To reduce fitting uncertainties and minimize the number of parameters, two correlations were applied to the fitting model: (1) as the spectra represent the gradual change of different species under different potentials, peaks corresponding to the same species across different spectra were constrained to have the same peak center position; (2) all peaks shared the same full width at half-maximum (fwhm), defined as , assuming the broadening of the FTIR features is consistent under the experimental conditions. Regarding parameter boundaries, the minimum allowable amplitude of each peak was set to zero, permitting the absence of particular species under certain conditions. The minimum peak center distance between the centers of neighboring peaks was constrained to be equal to or greater than 2σ, ensuring that the peaks do not overlap excessively and remain individually identifiable within the fitting model. Initial guesses for the peak center positions were configured using literature-reported values to minimize iterations during the fitting process.^88−90^ Additionally, the total number of peaks in the fitting model was optimized from 5 to 12 following the aforementioned fitting protocol, using the same constraints for peak fwhm, peak center positions, and peak distances. For each fitting model, the standard error (StdErr) and the median absolute deviation (MAD) of the residuals were evaluated. It was determined that 6 or 7 peaks provided the optimal configuration, capturing all significant features in the experimental data without introducing excessive variables that could lead to overfitting. It is worth mentioning that fitting with a pseudo-Voigt peak shape was also conducted using similar parameters and constraint rules; the results suggested that the Lorentzian contribution to the peak shape is negligible. Therefore, to reduce the total number of parameters, Gaussian peaks were exclusively used for the peak fitting.

## 3 Results and Analysis

### 3.1 Proton Reduction by MoFe Nitrogenase-Modified Electrodes (MoFe_Au_)

**Figure 4:**
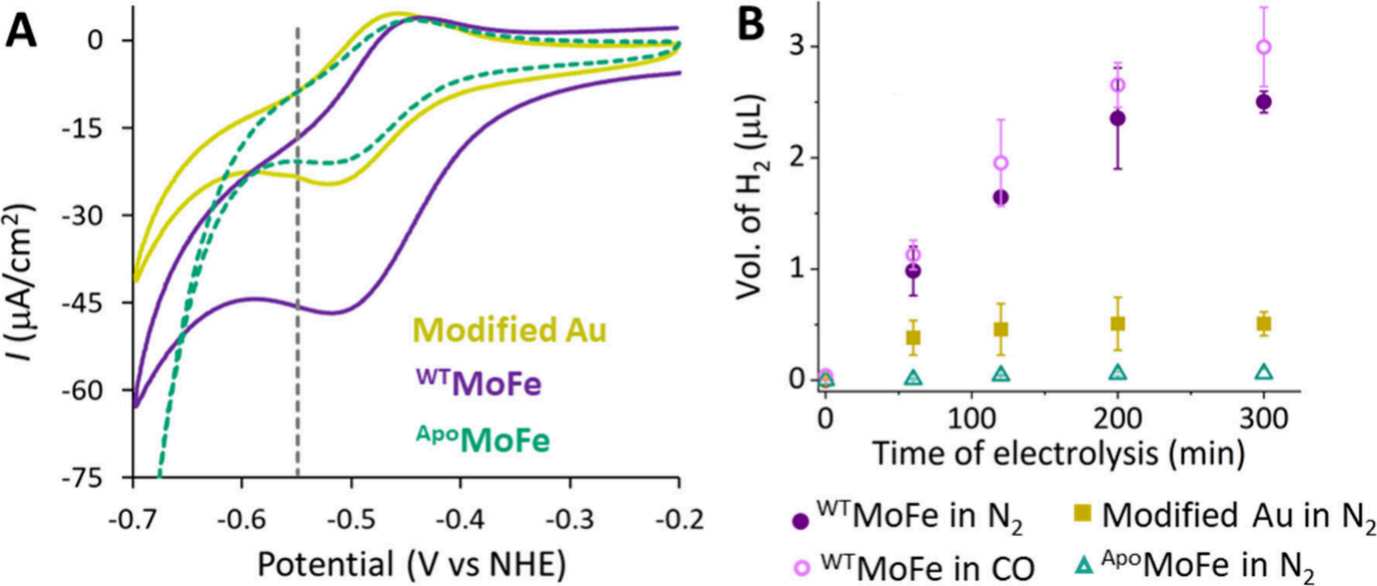
Cyclic Voltammetry data (A) and GC analysis of the headspace gas during bulk-electrolysis (B) of NBD-modified Au electrode without any protein (yellowish green), with ^WT^MoFe (violet), and with ^Apo^MoFe (dashed teal) in 100 mM MOPS pH 7 buffers, in the presence of 150 μM of MV mediator, in N_2_ environment using SCE and Pt wire as reference and counter electrodes, respectively. For CV the scan rate is 20 mV/s. In (B) the GC analysis of the headspace gas during bulk-electrolysis of ^WT^MoFe_Au_ immobilized electrodes under CO environments is shown in pink. Potentials have been corrected against NHE. The electrodes were held at −0.55 V (as shown in the dashed gray line in (A)) during the bulk-electrolysis experiments. The error bars in (B) represents standard errors.

The gold electrodes were modified with the WT or apo MoFe protein as discussed in a previous section. In order to evaluate the electrochemical activity of the protein’s active site, cyclic voltammetry (CV) was employed as shown in Figure 4A. Notably, the CV experiments were conducted in the presence of methyl viologen (MV) as a mediator, facilitating a homogeneous electron transfer from the electrode to the immobilized protein. This can be advantageous for spectroelectrochemical studies (see later sections) since it maximizes electron transfer to the majority of the immobilized protein. In contrast, in a direct electron transfer configuration (DET), unless the enzyme is ideally oriented on the electrode surface to allow DET to the majority of the sample, the protein’s different conformations and orientations on the electrode surface may result in mixtures of redox states or in a low redox control yield over the sample. Although the redox potentials of the metal clusters in the protein can vary upon covalent attachment, based on existing literature, the redox potential of MV (∼−0.47 V vs NHE in pH 7) lies between the redox potential of the P-cluster and that of the cofactor.^8,55^ This intermediary redox potential should increase the possibility of electron transfer from the electrode/mediator to the cofactor occurs via the P-cluster, aligning with our aim to emulate the WT protein’s behavior.

In the CV experiment, a potential sweep from positive to negative potentials in a pH 7 buffer under an N_2_ environment was performed. The results indicated a distinct catalytic current in the case of the WT protein (^WT^MoFe_Au_) (Figure 4A, violet), compared to the modified electrode without any protein (Figure 4A, yellowish green) where the nonturnover redox behavior of MV was only observed. This observation suggests that the protein retains catalytic activity upon attachment. To further confirm that the catalytic activity stems from the cofactor itself, similar experiments with the Apo version of the protein (^Apo^MoFe_Au_), which contains the P-cluster but lacks the cofactor, were conducted. As highlighted in Figure 4A (in dashed teal), the results show only the redox nonturnover signal from the mediator, similar to what was observed for the modified blank electrode. This result is intriguing, since literature reports of similar experiments utilize mediators with more negative redox potentials. In previous studies,^52,54,56,59^ the authors argued that their choice is influenced by the requirement of mediators with negative redox potentials to match the cofactor’s redox potential for nitrogen reduction, which is generally negative. Moreover, MV is not a competent reductant for MoFe in solution or for MoFe adsorbed on the electrode, suggesting that covalent attachment may play an essential role by inducing a conformational change that enables facile ET, as might have been the case when immobilized via a coordinate interaction on a His-tagged MoFe.^65^ This suggests that covalent attachment may induce conformational changes around the metal cluster, which is more pronounced upon application of negative potentials (*vide infra*), possibly making it accessible for facile ET, similar to what occurs during native enzyme activity.

To directly characterize the product, we then performed bulk electrochemistry experiments (Figure S3), holding the potential of the modified electrodes at −0.55 V vs NHE for a significant duration (∼5 h) in a closed electrochemical cell. The headspace gas was analyzed using gas chromatography (GC), which revealed the formation of hydrogen (Figure 4B). There was background H_2_ production from the modified blank electrode (Figure 4B, yellowish green squares), but at a much lower concentration compared to ^WT^MoFe (Figure 4B, solid violet circles). It is interesting to note that, with the ^Apo^MoFe-modified electrode (Figure 4B, open teal triangles), there was virtually no detection of hydrogen, in comparison to the modified blank electrode. This can be attributed to the presence of proteins (^Apo^MoFe and also, we believe, ^WT^MoFe in this case) on the electrode likely blocking any exposed Au electrode surface that might remain after chemical/electrochemical modification. To further confirm the behavior of the covalently attached catalytic protein, its reactivity was examined under different conditions, including in the presence of carbon monoxide (CO). As discussed earlier, while CO is an inhibitor of N_2_ reduction, MoFe nitrogenase is known to still reduce protons under CO conditions with high electron flux. In the bulk electrochemistry experiment, when CO was present instead of nitrogen, the production of hydrogen was observed (Figure 4B, open pink circles), demonstrating a behavior similar to native MoFe.

### 3.2 SEIRA-SEC on MoFe Nitrogenase-Modified Electrode

#### 3.2.1 Observation of S–H under Turnover Condition

**Figure 5:**
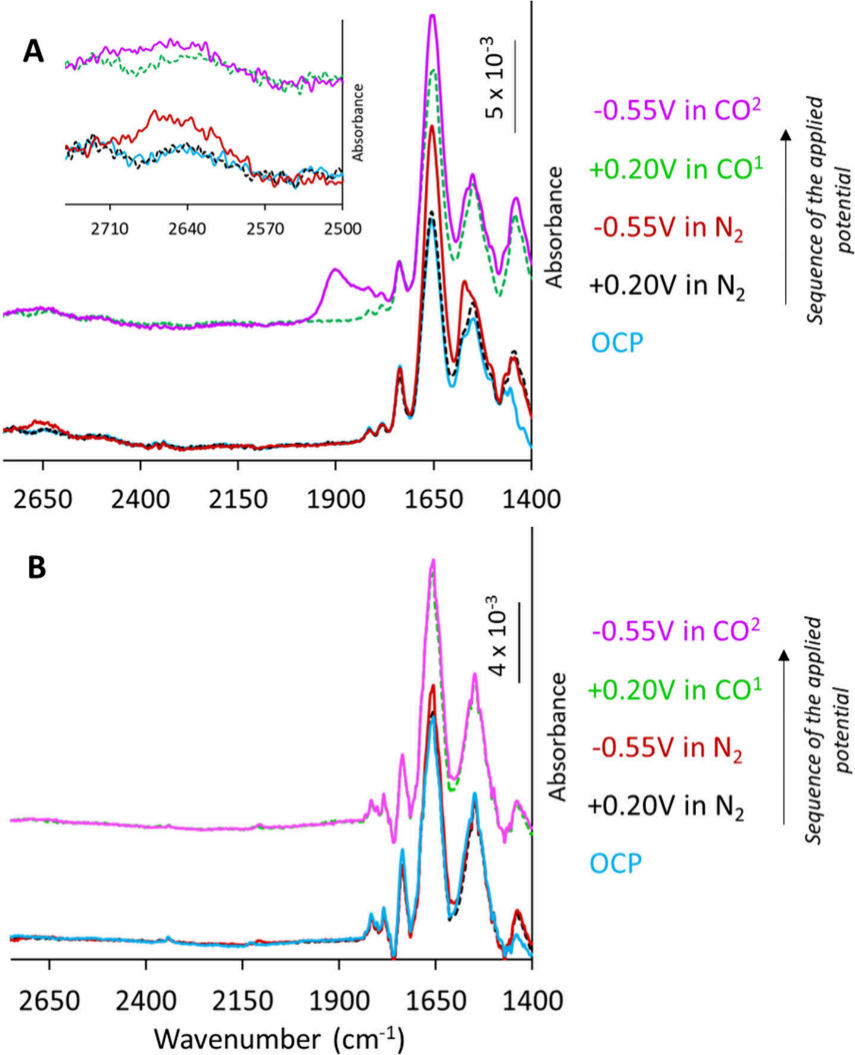
SEIRA spectra of ^WT^MoFe (A) and ^Apo^MoFe (B) immobilized on NBD-modified nanostructured Au electrode measured at different potentials in 100 mM MOPS pH 7 buffers containing 150 μM of MV mediator, in N_2_ or CO environment (as stated in the figure legend). *Note*: when in the CO environment condition 1 was done before 2. *Inset A*: Zoomed in the region 2750–2500 cm^–1^.

After establishing that ^WT^MoFe_Au_ is active and behaves like the native enzyme in its reactivity for H_2_ production (in both the presence and absence of CO), we aimed to observe this active site under turnover conditions using spectroscopic techniques. The method used for this experiment was Surface-Enhanced Infrared Absorption Spectroscopy (SEIRA), coupled with electrochemistry. To prepare the electrode, ^WT^MoFe was covalently attached to a modified nanostructured Au layer, as described in sections 2.2 and 2.5. Figure 5A presents the IR spectra of ^WT^MoFe_Au_ at the OCP (open circuit potential) and different applied potentials in pH 7 buffer under different environments. Upon the application of a positive nonreductive potential of 0.2 V (also the OCP) vs NHE under a nitrogen environment, no significant changes were observed in the spectra in comparison to the spectra observed before the application of potential (Figure 5A, sky blue vs dashed black spectra). However, when a potential of −0.55 V (where the protein shows catalysis) was applied, significant changes appeared at the amide stretches (∼1650 and 1570 cm^–1^), as well as in higher frequency regions (above 2600 cm^–1^) (Figure 5A, red). As mentioned earlier, the amide stretches arise from the protein’s secondary structure. This suggests that applying a negative potential, which provides electrons to the metal clusters, may induce conformational changes in the protein’s secondary structure. In the higher frequency regions of the IR spectra, we observed the emergence of a new (and broad) stretch between 2600 and 2700 cm^–1^. Repeating these experiments with the ^Apo^MoFe_Au_ showed no significant changes, apart from slight alterations in the amide regions (∼1650 cm^–1^, Figure 5B). This observation confirms ET to the ^Apo^MoFe_Au_, specifically to the P-cluster, and the lack of observable changes in the higher frequency region of ^Apo^MoFe_Au_, indicates that in ^WT^MoFe_Au_ these modulations (∼2650 cm^–1^) are related to a process at the FeMoco cluster.

The same experiment was conducted under different potentials, but in the presence of CO instead of N_2_. Under an oxidizing potential, the spectra resembled that observed under N_2_ (Figure 5, dashed green). However, at the reductive potential with ^WT^MoFe_Au_, in addition to the amide region changes and the appearance of the stretch around ∼2650 cm^–1^, an entirely new vibration was observed around ∼1900 cm^–1^ (Figure 5A, pink). The lack of both of these bands when repeating the experiment with ^Apo^MoFe_Au_ further confirmed that the observed vibrations in the ^WT^MoFe_Au_ originate from changes occurring at the cofactor site (Figure 5B, pink).

**Figure 6:**
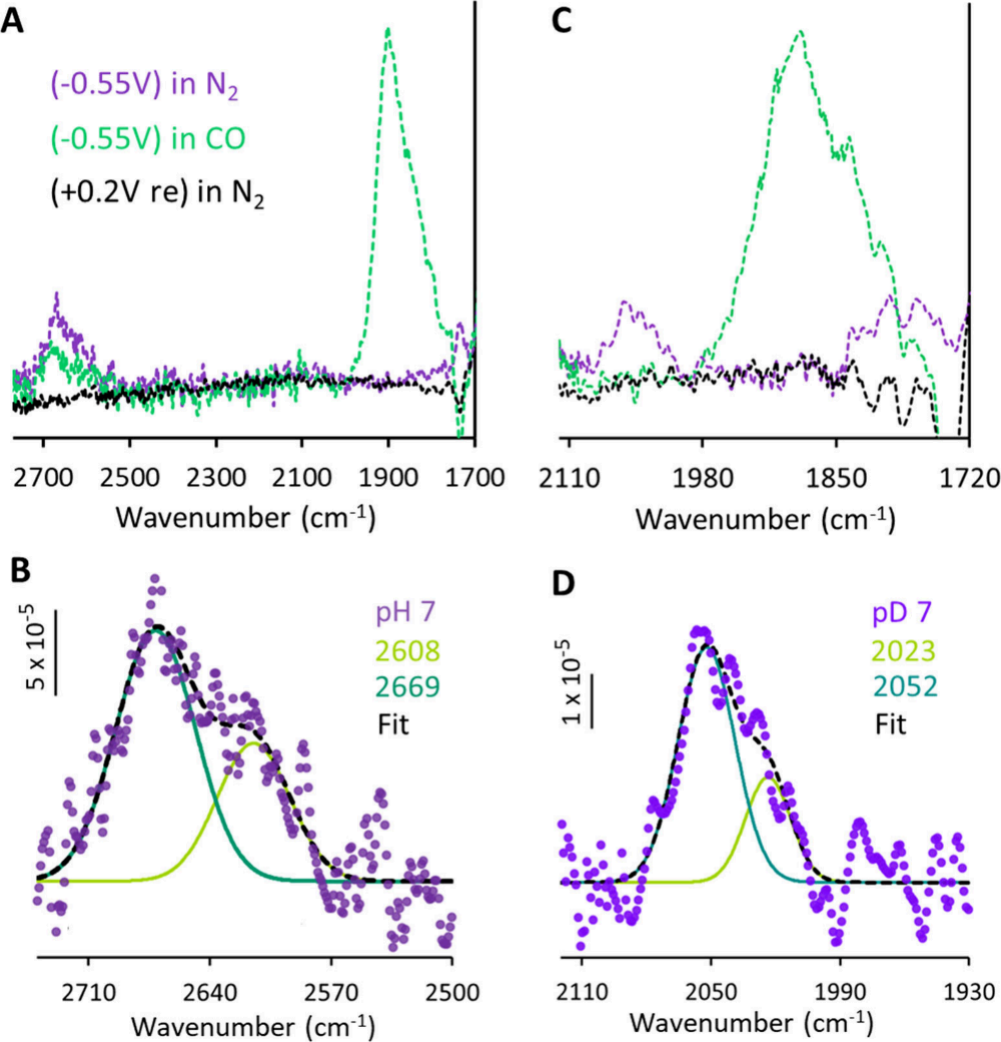
(A, C) Difference spectra of the SEIRA spectra (of ^WT^MoFe immobilized on NBD-modified nanostructured Au electrode) collected at different potentials under different conditions (N_2_ and CO) from the first spectra collected at OCP measured under N_2_ environment in 100 mM MOPS pH 7 (A) and corresponding pD 7 (C) buffers containing 150 μM of MV mediator. “re” refers to the spectra remeasured at 0.2 V under N_2_ environment, after CO unbinding. (B, D) shows the difference spectra (the spectra shown in A and C, respectively) at −0.55 V under N_2_ and their fitted components.

Figure 6A shows the difference spectra of the IR spectra collected at the reducing potentials in N_2_ (in violet) and CO (in green) environments, from the spectra collected initially at the positive potential of 0.2 V (which is the OCP). Differences in the stretch in the high frequency region are clearly visible in this figure, with the stretch appearing to be of lesser intensity, and also centered around higher frequency in CO compared to that in N_2_. This stretch is particularly broad (∼150 cm^–1^), suggesting the involvement of multiple species. We fit the spectra with multiple components, successfully resolving at least two components centered around 2608 and 2669 cm^–1^, with the peak at the higher frequency having more intensity (Figure 6B). To understand the origin of this particular stretch, we performed parallel experiments in pD 7 buffer. The observations were similar to those in the pH 7 buffer under the same conditions and the same applied potentials, the only exception being the stretching frequency in the higher energy region which shifted by approximately 600 cm^–1^ (Figure S4). The stretch is also broad (∼100 cm^–1^), although less broad compared to pH 7, and can also be resolved into two components centered around 2023 and 2052 cm^–1^ (Figure 6C,D and Figure S4). Although these components follow the trend that one would expect for the respective S–H/S–D shift, it is important to exercise caution when fitting multiple components under pD 7 conditions due to the low signal-to-noise ratio. Interestingly, the intensity of the stretch in pD 7 condition is decreased, which might be a result of the different dipole moments. This might also explain the lack of observation of the S–D stretch upon CO binding, as it will exhibit lower oscillation intensities, similar to what was observed under pH conditions. Comparing these findings to literature reports,^91,92^ the stretches in the 2600–2700 cm^–1^ may be assigned to S–H stretches, which could be formed during catalytic H_2_ production. We hypothesize that the higher frequency mode could correspond to a terminal S–H at FeMoco while the lower frequency mode may correspond to a bridging S–H. The correlation of the observed stretches to QM/MM calculations is discussed further in the theory section. We note that while protonation of sulfides in FeMoco has been amply discussed in the literature,^36,37,93−95^ the S–H stretches of FeMoco have never been previously observed.

#### 3.2.2 Understanding CO Binding to MoFe_Au_

**Figure 7:**
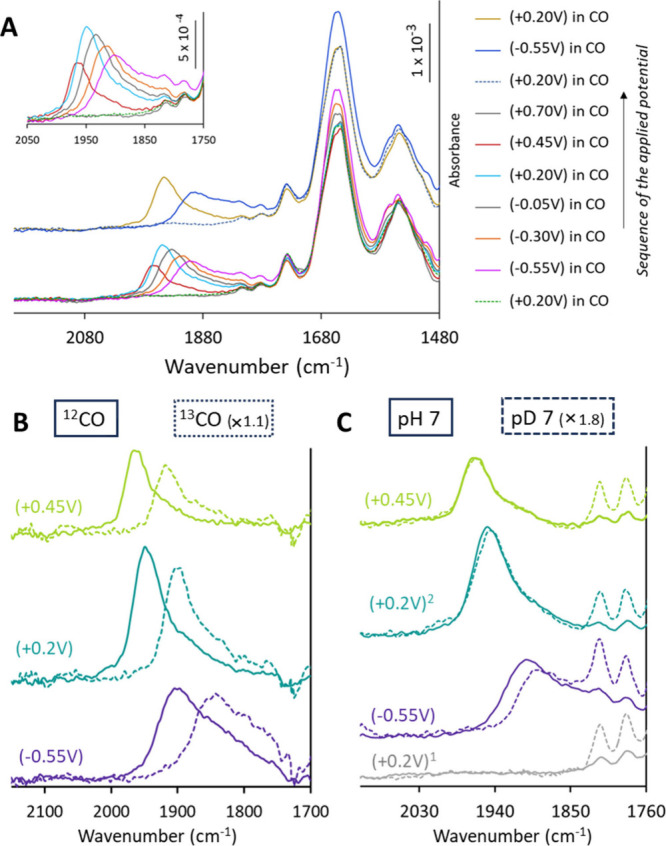
(A) SEIRA spectra of ^WT^MoFe immobilized on NBD-modified nanostructured Au electrode collected at different potentials under CO environment in 100 mM MOPS pH 7 buffers containing 150 μM of MV mediator. (B) Difference spectra of the SEIRA spectra as A measured under ^12^CO (solid lines) and ^13^CO (dashed lines) from the corresponding SEIRA spectra measured under OCP in N_2_. (C) SEIRA spectra as measured under ^12^CO in pH 7 (solid lines) and pD 7 (dashed lines) buffers. Condition 1 was performed before CO binding (i.e., before −0.55 V), and condition 2 after CO binding.

As previously mentioned, under resting state conditions or at OCP in pH 7, in the presence of CO, there were no observable changes in the IR spectra of our ^WT^MoFe_Au_. Changes were only observed at negative potentials (−0.55 V), where catalysis occurs, including the appearance of new vibrations around 1900 cm^–1^ (Figure 5, Figure 7A). This observation is consistent with existing literature, which indicates that molybdenum nitrogenase does not bind CO at the resting state and requires turnover conditions to bind CO (i.e., it must be reduced beyond E_0_).^34,96^ In order to confirm that the observed stretch indeed originated from CO binding to the cofactor, we conducted the same experiment under similar conditions using ^Apo^MoFe_Au_. In the presence of CO, no new peaks appeared in the spectra of ^Apo^MoFe_Au_ (at any potentials) (Figure 5B), confirming that the broad stretch observed in the ^WT^MoFe_Au_ experiments was indeed due to CO binding to the cofactor.

Having the protein covalently attached to the electrode, and omitting the use of the reductase protein or the regeneration system during the experiment, not only allowed us to bypass the iron-protein cycle but resulted in a much cleaner system, where simply altering the potentials enabled us to transition between reductive/catalytic and noncatalytic conditions. By shifting to higher potentials, we could begin oxidizing the metal clusters. This setup inspired us to study CO unbinding from the cofactor, a topic less explored in the literature. Upon moving to higher potentials, after binding CO at −0.55 V (Figure 7A, pink), the broad stretch sharpens and shifts to higher energy with increasing positive potentials (from −0.30 to +0.45 V, Figure 7A, orange to red), and eventually disappears at a significantly positive potential (+0.70 V, Figure 7A, gray) compared to the OCP or the initial starting potential. This change in the CO vibration upon sweeping to positive potentials can be explained by the oxidation of the metal cluster, specifically the metal centers. As the cofactor becomes relatively more oxidized, the CO stretch frequency increases due to decreased back bonding, which shifts it to a higher frequency. Our observations highlight unique aspects of CO interaction with the cofactor under varying oxidation states. It suggests that while CO cannot bind at the resting state (E_0_), once it is bound during turnover conditions, it may stay bound even in the E_0_ state and only releases upon further oxidation of the FeMoco. To further confirm that these peaks originate from CO binding, all experiments were also conducted using ^13^CO instead of ^12^CO. These experiments yielded very similar observations, with a notable shift of approximately 45–47 cm^–1^ in the C–O stretch (Figure 7B), as has been previously reported for ^13^CO.^88,89^ It is noteworthy that the process of binding and unbinding CO can be repeated multiple times on the modified electrodes, indicating the stability of the protein on the electrode (Figure 7A), as also observed during previous bulk electrolysis experiments (Figure S3), further emphasizing the robust nature of our experimental setup and the stability of the protein-electrode interaction.

Although the stretching frequency is in the range that might be expected for CO binding to the FeMoco from known literature, it is significantly broader (∼120 cm^–1^) than what has been observed before (∼30 cm^–1^),^88,89^ an exception being a recent study of MoFe on electrodes (∼110 cm^–1^).^56^ This may be attributed to the presence or accumulation of multiple CO-bound intermediates in our experiments (see Discussion section for further details). Thus, under catalytic conditions, the stretch appears broader, as the cofactor is in constant turnover, supported by the generation of H_2_ in our bulk electrolysis experiments (Figure 4B, pink), which will populate a range of intermediates. The stretch becomes sharper when we move away from catalytic potential, as it will lead to the decay of the intermediates (*vide infra*). To further understand CO binding dynamics to nitrogenase, we conducted similar experiments using pD 7 buffer instead of a pH 7 buffer. Two significant differences were observed. First, the CO binding process was comparatively slower in pD 7, as indicated by a gradual increase in the CO vibrational frequency over time when the electrode was held at the catalytic negative potential of −0.55 V (Figure S5). Second, and intriguingly, only under turnover conditions (e.g., at potential −0.55 V) the observed stretch shifted to lower energy by 10 to 12 cm^–1^ in comparison to the corresponding pH 7 spectra (Figure 7C, violet spectra). However, as the potential was increased away from the catalysis potential (Figure 7C, teal, and yellowish green spectra), essentially no shift was observed. These observations strongly suggest the involvement of an exchangeable H/D, which affects CO binding and its vibration, in particular under catalytic conditions.

A unique observation to highlight at this stage is the lack of any distinct feature (no clear shift in ^12^CO vs ^13^CO) that could be assigned to the C–O stretching frequency of a bridging CO, which should appear at ∼1700 cm^–1^ (Figure S6). Previous literature, based on both spectroscopic analyses and crystal structures, has reported the presence of a bridging CO during CO binding studies.^35,89^ However, under our steady state turnover conditions, we did not observe such a feature. This could be due to the possibility of the bridging CO stretch overlapping with the protein amide and the carbonyl stretches of the NHS ester regions, which might have masked its detection. However, it is also possible that under the high electron flux conditions utilized in our experiments, no bridging CO is formed.

### 3.3 QM/MM Calculations

**Figure 8:**
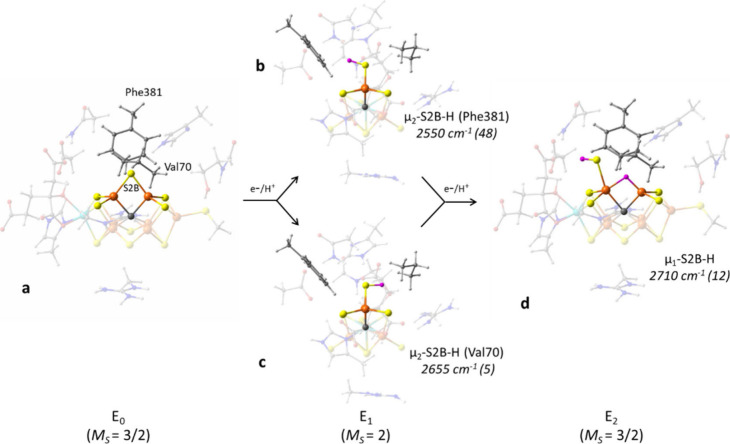
Proposed mechanism for the addition of e^–^/H^+^ to the FeMoco based on QM/MM calculations. The first two early intermediates (E_1_ and E_2_) have been considered in this figure. The calculated vibrational frequencies are mentioned in the figure along with the stretching intensity (km mol^–1^) in parentheses. The atoms of the FeMoco are colored as following; Fe = orange, S = yellow, C = gray and H = magenta.

To better understand the origin of the two observed components in the S–H stretching region (Section 3.2.1), a series of QM/MM calculations were performed. Models were tested for a range of protonated E_n_ models as summarized in Table 1 and shown in Figures 8, S7, and S8. Overall, the calculations show S–H stretches in the 2550–2720 cm^–1^ range with S-D stretches shifting to the 1830–1960 cm^–1^ range. The S–H stretches are in excellent agreement with the experimentally observed range. However, the S–H/S-D shifts are somewhat overestimated and may reflect the fact that the harmonic frequency calculations do not capture anharmonicity and mode coupling, which may differ significantly. Additionally, it should be noted that an exchange between H_2_O/D_2_O, may also lead to changes in residue conformation and subsequently affect H-bonding with S–H vs S-D in the experiments, which cannot be taken into account in calculations. Nevertheless, the general agreement provides a context to discuss the range of possible protonated E_n_ models.

**Table 1:** Calculated and Experimental S–H/S–D Stretching Frequencies for Models of FeMoco Redox States^a^

State	S–H, cm^–1^ (Intensity, km mol^–1^)	S–D, cm^–1^	Experimental observation, cm^–1^
E_1_(μ_2_-S2B-H-Val70)	2655.8 (5.1)	1908.3	S–H frequencies = 2608, 2669
E_1_(μ_2_-S2B-H-Phe361)	2549.4 (48.1)	1831.8	
E_2_(2,6-(μ_2_-H)/6-μ_1_-SH)	2711.4 (11.1)	1948.7	
E_3_(μ_1_-2-H_2_/6-μ_1_-SH)	2706.7 (2.9)	1945.5	S–D frequencies = 2023, 2052
E_3_(2,6-(μ_2_-H)_2_/6-μ_1_-SH)	2719.2 (3.1)	1954.5	
E_3_(2,6-(μ_2_-H)/2-μ_1_-H/6-μ_1_-SH)	2723.0 (8.2)	1956.9	

a Note: the states are defined by mentioning the Fe center where the specific ligand is present. For example, E_2_(2,6-(μ_2_-H)/6-μ_1_-SH) indicates a bridging hydride at Fe2 and Fe6 and a terminal SH at Fe6. Figures of the models are shown in Figure 8 and in the SI, Figures S7 and S8.

Previous experimental and computational studies in our group have suggested that the first protonation event occurs at the S2B bridge in the E_1_ state of FeMoco (the S5A bridge was also noted as a possibility).^26^ Upon protonation of the 1e^–^ reduced FeMoco, the belt-sulfide S2B can orient its S–H bond toward either the Val70 (Figure 8c) or the Phe381 (Figure 8b) residues. The calculations revealed that both conformations are nearly degenerate, with the S–H bond preferentially directing toward the Phe381 residue by only 0.9 kcal/mol. Despite their near-degenerate energies, the calculated S–H vibrational frequency for the Phe381 conformation (2550 cm^–1^) appears to be red-shifted compared to the Val70 conformation (2655 cm^–1^). We attribute the large difference in the S–H vibrational mode between the near-degenerate E_1_ conformations to their protein environment. Our QM/MM structures suggest an S–H/π interaction between the S–H and aromatic Phe381 group. The analogous interaction has recently been discussed with respect to Ryde and co-workers’ QM/MM study of the Fe-only N_2_ase’s E_1_ state.^97^ The red-shift and increased intensity of S–H vibrational modes, resulting from S–H/π interactions have been illustrated in organic thiol systems.^91^

With the further delivery of e^–^/H^+^, for instance, when it reaches the E_2_ state, the coordination mode of the belt sulfide can change from a μ_2_-bridging to a terminal mode, accompanied by protonation. We have previously assigned the lowest energy E_2_ isomer with our QM/MM protocol to adopt a terminal SH^–^ group at the Fe6 site to facilitate μ_2_-hydride (μ_2_-H^–^) formation (Figure 8, d).^37^ We would expect a small torsional barrier associated with the rotation of the terminal SH^–^ group in relation to the μ_2_-bridging conformation, but with similar vibration frequency which might contribute to the broadening of experimental stretches (Figure S7). Our calculations indicate that the vibrational frequency of the terminal SH^–^ increases to 2700–2710 cm^–1^ compared to the SH^–^ stretch in the bridging mode at E_1_ (2550/2655 cm^–1^). The terminal SH^–^ group preferentially directs toward an anionic carboxylate group of the homocitrate ligand than the μ_2_-bridging hydride (1.8 kcal/mol). Comparing the vibrational frequencies obtained from the calculations to our experimental data, we suggest that the two experimentally observed frequencies from the SH^–^ vibration (Section 3.2.1) correspond to different configurations of the SH group, likely in different redox states. The lower frequency is most likely associated with a protonated bridging sulfide (perhaps in the E_1_ redox state), while the higher frequency, which is more intense than the lower frequency, corresponds to a terminal SH^–^ (in a more reduced state, perhaps E_2_ or beyond). Furthermore, the experimentally observed frequency difference between a terminal and bridged thiol is approximately 61 cm^–1^, which is in close agreement with the QM/MM calculated difference of 55 cm^–1^ in consideration of the orientation of the μ_2_-SH^–^ bond toward Val70 (Table 1). It is interesting to note that the calculations also indicate the terminal SH mode in the E_2_ state has approximately twice the vibrational intensity of the bridging E_1_ conformation (Figure 8, Table 1), which appears consistent with our experimental observations (Figure 6). Another possible explanation for the higher intensity of the terminal SH stretch is the presence of multiple species with terminal SH groups with similar stretching frequencies. This is directly observable in the calculations for E_3_,^38^ where the terminal SH, depending on the different conformations that E_3_ can adopt (Figure S8), has a stretching frequency very similar to that of the terminal SH in E_2_ (Table 1).

## 4 Discussion

Our experiments with MoFe nitrogenase-modified electrodes (^WT^MoFe_Au_) demonstrate stable protein attachment and significant catalytic activity, as evidenced by cyclic voltammetry and gas chromatography results. The choice of methyl viologen as a mediator, despite its more positive redox potential, effectively facilitated electron transfer, suggesting that conformational changes likely occur upon immobilization of the protein via covalent attachment. The modulations in the amide bands as a function of applied potential provide further experimental support for this hypothesis. To further test this hypothesis, parallel experiments were conducted in which CO binding to ^WT^MoFe was studied via ATR spectroscopy using chemically reduced methyl viologen. However, in those control experiments, no CO binding was observed (Figure S9), suggesting that in solution, or in such simple drop-casted conditions, FeMoco, in absence of FeP, is not reduced by methyl viologen. This observation is consistent with a previous literature report, where MoFe adsorbed on an electrode could not be reduced by low potential mediators.^63^

The strategy employed herein aimed to emulate the electron transfer characteristics of the native enzyme, thereby providing a model system that more accurately reflects natural enzymatic conditions, while bypassing the rate-limiting FeP cycle. The significant catalytic currents observed with the ^WT^MoFe protein compared to the minimal activity with the ^Apo^MoFe and long-term stability underscore the potential of our system to serve as a platform for further spectroelectrochemical studies enabling us to understand key features of the mechanism.

The integration of SEIRA with electrochemistry (SEIRA-SEC) enabled the observation of the covalently attached enzyme on a monolayer under redox control. The application of a negative potential, where we observe H^+^ reduction, led to significant changes in the amide stretches, suggesting alterations in the protein’s secondary structure possibly linked to the electron flow through the protein’s metal clusters. Along with the amide band modulations, there was the emergence of a new, broad stretch between 2600 to 2700 cm^–1^ in pH 7 (which shifted by ∼600 cm^–1^ in pD 7) (Figure 5 and Figure 6) and that was assigned to S–H stretches of the FeMoco cluster. During catalysis, protons and electrons are sequentially delivered to the cofactor. While it is generally agreed that the early ET processes occur at the iron site of FeMoco, the fate of the H^+^ is a matter of debate, with the possibility of hydride and/or protonation of the belt sulfides in FeMoco being possible at various E_n_ states. Indirect support for belt sulfide protonation has come from combined EXAFS and QM/MM studies,^26^ and also from ^2^H Mims ENDOR studies,^32^ but until now there has been no direct spectroscopic evidence for the reversible formation of both bridging and terminal S–H groups at FeMoco during turnover conditions. The current observations provide direct experimental support for this hypothesis and align with predictions regarding the role of sulfides in nitrogenase catalysis. Previous crystallographic and theoretical studies have demonstrated the lability of the belt sulfides. Crystallographic studies have shown that belt sulfides like S2B can be directly replaced by CO^34,35^ or by a light-atom ligand (likely OH) in V nitrogenase^98^ under certain conditions. Alternatively, theoretical studies have suggested that protonated sulfides can assume a terminal position, in reduced or inhibited states, either because of the presence of hydride or CO in a bridging configuration.^36,45^ Our theoretical calculations have provided further insights, indicating that an S–H stretch in the bridging configuration, when the sulfide is bonded to two metals, exhibits distinct vibrational frequencies with distinct frequencies compared to those in a terminal S–H configuration (Section 3.3, Figure 8). Thus, under our steady state turnover conditions, we observe at least two components at 2669 and 2608 cm^–1^ under pH 7, relevant to the mechanistic cycle. Comparing theoretical calculations and experiments, it can be reasonably argued that under our experimental conditions, the bridging SH is directed toward the Val70 residue, a residue that has been discussed in the literature as an important component involved in the Mo–N_2_ase catalytic cycle.^99,100^ It is noteworthy that QM/MM calculations also support the experimental observation where a terminal SH has a higher vibrational/oscillation intensity compared to a bridging SH. Although it is somewhat speculative, one could argue that the higher intensity for the terminal SH is due to the presence of multiple components with terminal SHs, as would be the case for any intermediate post E_1_ (Table 1). Upon CO binding, our results indicate that CO has triggered the opening of the bridging sulfur sites to a terminal position (irrespective of the intermediate state) and also perturbed the electron density of the active site, making it relatively oxidized (as expected), which is reflected in the lower intensity and higher frequency of the S–H vibration (Figure 6A).

**Figure 9:**
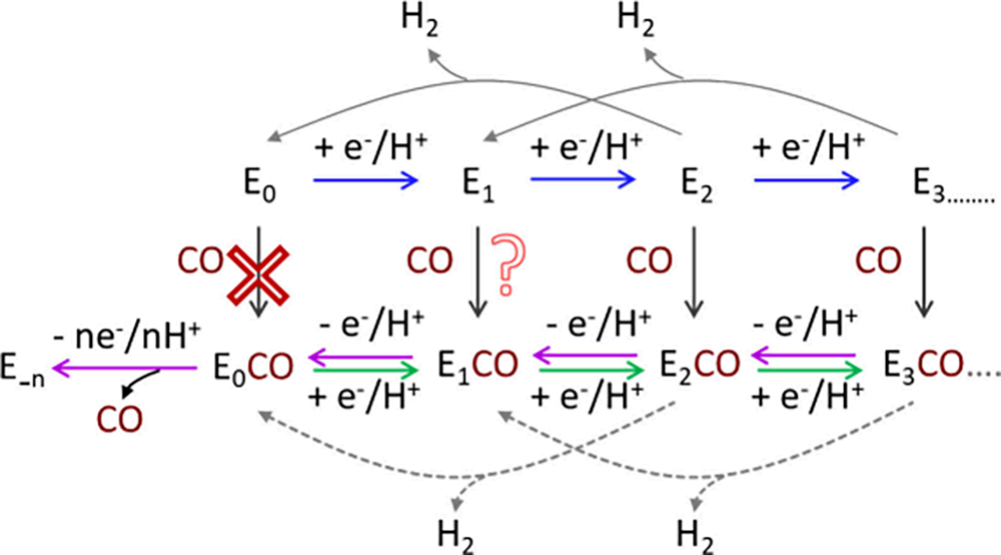
Schematic presentation of probable H_2_ production mechanism under N_2_ and CO environment.

As highlighted in the introduction, studying CO binding dynamics, therefore, yields critical insights into the structural and functional adaptability of nitrogenase, especially for Mo–N_2_ase as CO acts as an inhibitor for N_2_ reduction but not for other substrates like H^+^, as also observed in our electrochemical experiments. Our spectroelectrochemical experiments have confirmed that CO does not bind to the resting state of MoFe, but requires the enzyme to be in turnover conditions (Figure 9, black arrows). We note, however, that based on these experiments, it is not possible to confirm the exact first intermediate state to bind CO (E_1_ or a later state). The reversible nature of CO binding is demonstrated by the shifts in IR spectra with changing potentials.

It is noteworthy that the N_2_ase CO stretches are inherently broader as observed in previous solution studies (peak base is about ∼30 cm^–1^)^88,89^ compared to the CO stretches from other proteins like hydrogenase. This broadening may be attributed to the distinct hydrogen bonding networks present at the MoFe active sites, which may lead to a broader distribution of frequencies. This phenomenon is well-documented in the literature for myoglobin.^101^ Studies have shown that mutations around the metal active site can result in a broadened CO stretch when CO is bound to the myoglobin active site. However, in this study, the CO stretches are even broader (∼120 cm^–1^, Figure 7). The broadness of the CO stretch in our experiments can be further attributed to the presence of multiple species under turnover conditions. As previously mentioned, literature reports have documented multiple IR stretches originating from CO binding to MoFe-N_2_ase in solution. However, those conditions require the interaction of the Fe-protein with ATP and MoFe, which imposes diffusional limitations on the experiment, which may slow down processes, and in turn, generate a narrower distribution of intermediates over their reaction time.^88,89^ Under our spectroelectrochemical conditions, where electrodes are held at a constant potential, ^WT^MoFe_Au_ undergoes multiple turnovers due to a steady electron supply. The persistence of a relatively wide distribution of intermediates forming on the electrode surface in the presence of CO can be attributable to the continued catalytic activity of the protein in reducing H^+^ even under CO exposure. This observation can be elucidated by adapting the Lowe-Thorneley cycle to include the influence of CO (Figure 9). This leads to the presence of intermediates capable of binding CO, resulting in a broader CO stretch at reducing potentials. These intermediates (E_2_CO, E_3_CO, . . .) could revert to earlier stages in the cycle, producing H_2_, similar to those suggested with the regular cycle (E_2_, E_3_, . . ., Figure 9), and capable of further reduction (Figure 9, green arrows) thereby sustaining a continuous cycle of differing intermediate states. In contrast, as we shift to oxidizing or more positive potentials, fewer intermediates are accessible on our electrodes (Figure 9, pink arrows). Consequently, the peaks become sharper due to the decreased presence of a narrower distribution of more reduced intermediates. An intriguing finding from our studies, that is not readily explained, is the requirement of going beyond the E_0_ state to a more oxidized state (e.g., to positive potentials) in order for CO to unbind. We note, however, that this appears consistent with previous observations from Vincent and co-workers.^56^ While initially perplexing as CO is unable to bind a resting E_0_, it is essential to recognize that electronically, the states E_0_ and E_0_CO are distinctly different. Furthermore, it could be argued that the crystal structure of CO bound to the MoFe cofactor might represent a stable E_0_CO species rather than an intermediate prone to reversion (by releasing H_2_) under crystal growth conditions.

The inherent differences between previous solution experiments and our steady-state turnover conditions with CO might also explain the lack of any clearly observable C–O stretching frequency in the 1700 cm^–1^ region, which can be assigned to a bridging CO in the present experiments (Section 3.2.2). It is possible that under the comparatively lower flux conditions utilized in solution CO binding studies and for the crystallographic studies the MoFe protein tends to transition or relax into a more stable form which leads a terminal CO to bridge, compared to the dynamic state observed under steady-state turnover conditions. Notably, in crystal structures, it has been clearly demonstrated that the presence of a bridging CO coincides with the complete disappearance of the bridging sulfide.^34^ The reversibility of CO binding to MoFe is widely accepted. However, the complete elimination of the bridging sulfide seems unlikely with respect to reversibility or continuing H^+^ reduction. Instead, the absence of a bridging CO indicating the presence of an axial/bridging S–H group consistently bound to the active site provides a more plausible explanation for the reversibility of CO binding and unhindered H^+^ reduction during physiological turnover conditions. It is noteworthy that in our experiments, we simultaneously observe both the SH stretch and the CO stretch, under catalytic conditions, bound to the cofactor, which strongly supports that the sulfide bridge is not lost under steady state turnover.

In an effort to deconvolute the various components contributing to the broad CO vibration frequency, we employed a global fitting approach to all spectra collected at varying potentials using the minimal number of components necessary. This analysis successfully separated the spectra into six distinct components (Figure S10). By integrating the area under the curve for each component, a clear trend was observed, indicating changes in intensity with varying potentials (Figure S11). Components with stretching frequencies centered around 1920 and 1942 cm^–1^ (in pH7, 1913, and 1941 cm^–1^ in pD 7) were observed to initially increase in intensity, but then decrease as the potential shifted from positive to negative. These stretching frequencies are very similar to 1906 and 1936 cm^–1^ that were previously reported in the literature.^88,102,103^ A closer look at the stop-flow IR kinetics and the EPR-based experiments, and their experimental flux conditions, indicates that these frequencies are likely related to early CO-bound intermediates (e.g., at E_1_ and E_2_). Thus, under our experimental conditions, at more negative potentials, the 1920 and 1942 cm^–1^ components have lesser intensity, and the components with CO stretching frequencies ranging between 1890 and 1800 cm^–1^ gain intensity. Although similar frequencies (∼1880, 1858 cm^–1^) have been observed in the literature in very small amounts,^48,88^ their origin has not been clearly discussed. We speculate that the 1890–1800 cm^–1^ stretches may originate from later intermediates, such as post E_2_ and thus are more prone to decay under limited electron flux. Interestingly, under oxidizing/positive potentials, the CO stretch that grows in intensity is centered around ∼1961 cm^–1^ (both in pH 7 and pD 7). Similar stretches, although not assigned to a specific E_n_ state, have been observed experimentally where a stretch at 1958 cm^–1^ grew in intensity, monitored with stop-flow IR kinetics, while the CO stretches around 1906 and 1936 cm^–1^ decayed.^88^ Comparing to a previous QM/MM study where similar frequencies were observed in an E_0_-CO model, the experimental 1961 cm^–1^ stretch might be attributable to a CO bound in state with the E_0_ redox level.^45^ These results support our discussion that once CO binds during turnover, it does not unbind from E_0_ and requires higher oxidation to completely unbind. However, it is premature to further elucidate the exact nature of these components, specifically, the origins of individual CO stretch frequencies from their respective components remain indeterminate, highlighting the inherent limitations of infrared spectroscopy despite its sensitivity. This would need parallel spectroscopic techniques, which would enable looking directly at the metal centers to gain electronic information, which is beyond the scope of the present work. Nevertheless, CO binding-unbinding studies hold unique significance in nitrogenase research, as CO acts as an IR-sensitive ligand to the FeMoco, which inherently lacks specific IR-sensitive ligands. Therefore, employing CO in these studies provides critical insights into the underlying mechanisms. It is well established in the literature that among the intermediates formed during the turnover of MoFe N_2_ase, although studied without CO interference, hydrides play a role in many of the intermediates. There is substantial literature that has sought to elucidate the characteristics of hydrides, particularly their binding properties at the cofactors of nitrogenases.^6,13,31,32,104^ In the reaction mechanisms of Fe–Fe hydrogenases the involvement of a hydride is well-documented.^105,106^ During HD exchange studies, it has been observed that the CO ligand, positioned trans to the hydride at the active site, exhibits a shift in its IR stretch frequencies by approximately 7–12 cm^–1^.^107^ This observation mirrors our findings (Figure 7C), suggesting the involvement of hydrides in the cofactor in the CO bound intermediates, which might be expected because of uninhibited H_2_ production. This hypothesis is further supported by direct comparison of the results obtained from the global fitting of CO stretches under varying potentials in pH 7 and pD 7. Among the deconvoluted components, three stretching frequencies, 1852, 1889, and 1920 cm^–1^, in pH 7 exhibited a distinct shift under pD 7 to 1840, 1879, and 1913 cm^–1^, respectively, while other components remained almost unchanged (Figure S11, Table S1). This highlights that only a few of the intermediates likely harbored a hydride, as might be expected, capable of influencing a shift in the CO stretch frequencies upon H^–^/D^–^ exchange.

## 5 Conclusion

In conclusion, this study underscores the significance of covalently attaching MoFe nitrogenase to the electrode surface, a modification that enhances the robustness and efficacy of electrochemical measurements and enables us to go past the rate-limiting reductase protein cycle, while preserving the native-like behavior of the metalloenzyme. This approach allowed us to perform detailed spectroscopic investigations, which provide insights into the dynamic behavior of catalytic processes at the molecular level, particularly through the elucidation of intermediate states. Specifically, we provide direct experimental evidence for protonation of the bridging sulfides of FeMoco during steady state turnover conditions, demonstrating their direct involvement in the catalytic cycle of nitrogenases (under both CO and N_2_ conditions). Of particular interest is the fact that while both bridging and terminal S–H stretches are observed, the bridge is not lost during turnover, which is in contrast to hypotheses based on protein crystallographic data.^34,35^ The spectroscopic data derived from CO binding-unbinding experiments reveal critical information about mechanistic cycle of the enzyme, showing that CO can bind to a range of E_n_ states. The distinct shifts observed in IR stretching frequencies upon H/D exchange, indirectly indicate the involvement of bound hydrides in a subset of the CO-bound intermediates and further illustrate the potential of this methodology to probe subtle changes in ligand interactions. Importantly, the sensitivity of the approach utilized herein provides an avenue for understanding the mechanism not only of Mo nitrogenase, but also for the investigation of alternative nitrogenases. The exploration of a wider substrate scope to elucidate key mechanistic features of the nitrogenase family of enzymes is part of ongoing work in our research group.

## Supporting Information Available

The Supporting Information contains additional figures (and table) from the BE experiment, SEIRA data, Computational conformers, and fitting of the CO stretch. All relevant data generated and analyzed during this study, which include experimental, and computational data, will be made available in the Edmond Open Research Data Repository 10.17617/3.TAXLEE. (PDF)
