# Supplementary material for: Investigating the Molybdenum Nitrogenase Mechanistic Cycle Using Spectroelectrochemistry
Source: J Am Chem Soc. 2025 Jan 2;147(2):2099–114. doi: 10.1021/jacs.4c16047 (PMC11744760; doi:10.1021/jacs.4c16047)
Supplement: Supplementary file 1 — ja4c16047_si_001.pdf [file ja4c16047_si_001.pdf]

Supporting Information

Investigating the Molybdenum Nitrogenase mechanistic cycle using spectroelectrochemistry

Kushal Sengupta*, Justin P. Joyce, Laure Decamps, Liqun Kang, Ragnar Bjornsson, Olaf Rüdiger*, Serena DeBeer*

Department of Inorganic Spectroscopy, Max Planck Institute for Chemical Energy Conversion, Mülheim an der Ruhr, Germany, 45470

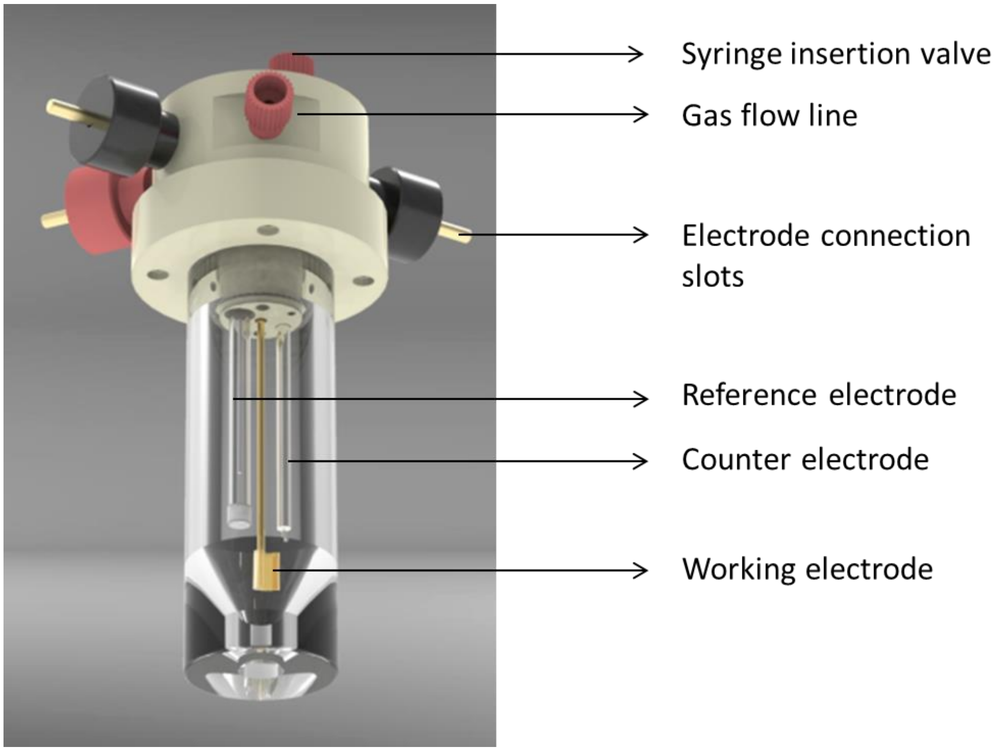

Figure S1. Air-tight electrochemical cell constructed at the MPI CEC, which was used for bulk electrolysis experiments. The RE and CE are separated into different compartments by two frits.

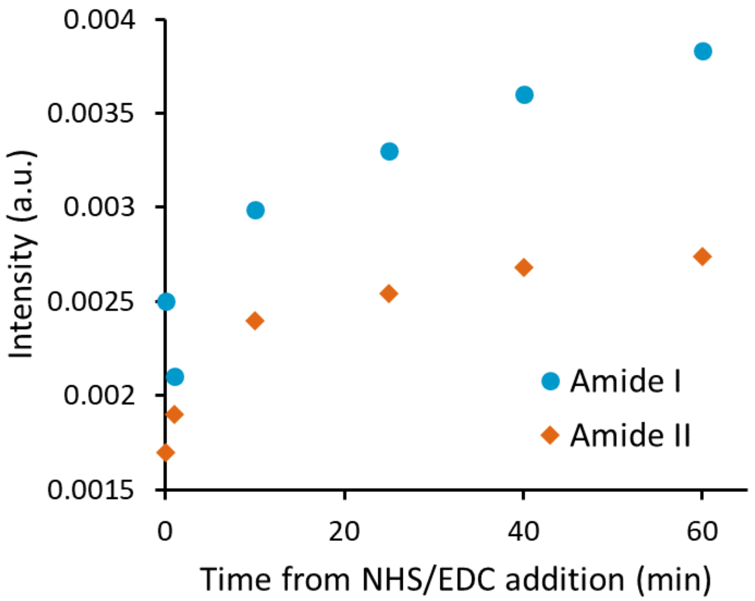

Figure S2. Intensities of the Amide I and Amide II stretches plotted against time (from Figure 3C) to show the gradual growth of the features followed by a saturation.

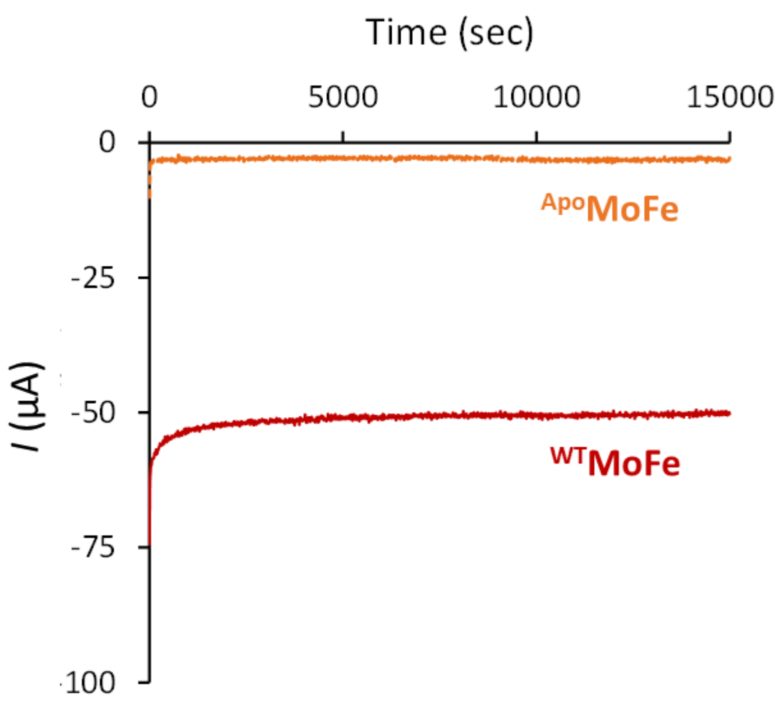

Figure S3. Bulk electrolysis (held at -0.55 V *vs* NHE) of 4-NBD modified Au electrode with ^{WT}MoFe (red) and with ^{Apo}MoFe (orange) with 150µM MV in 100 mM MOPS **pH 7** buffers in N_2 environment using Ag/AgCl and Pt wire as reference and counter electrodes, respectively.

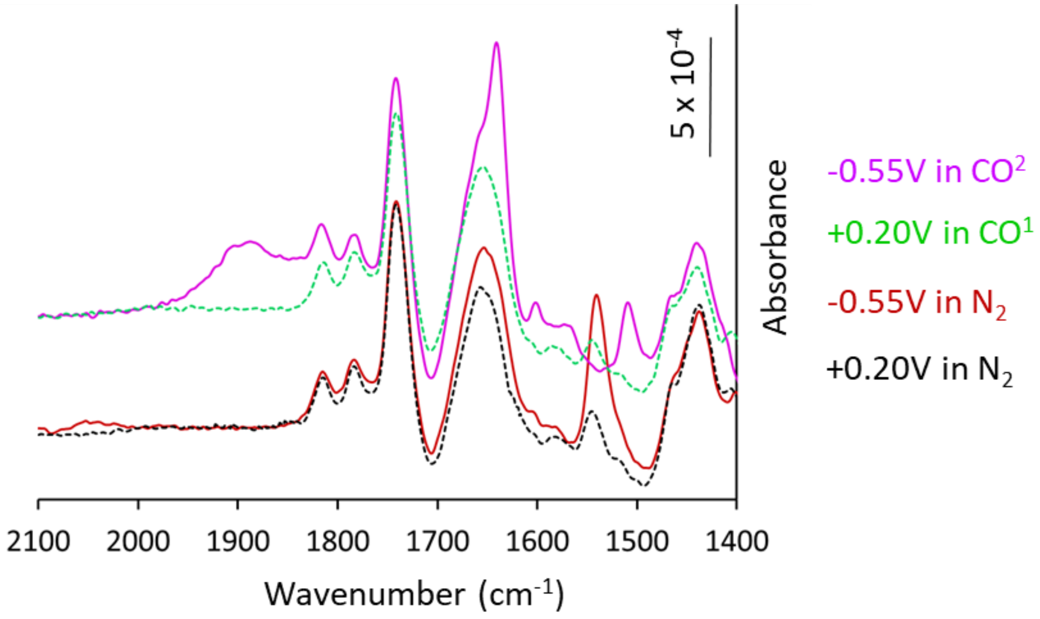

Figure S4. SEIRA spectra of ^{WT}MoFe immobilized on NBD-modified nano-structured Au electrode measured at different potentials in 100 mM MOPS **pD 7** buffers containing 150 µM of MV mediator, in N_2 or CO environment (as stated in the figure legend). Note: when in the CO environment condition 1 was done before 2.

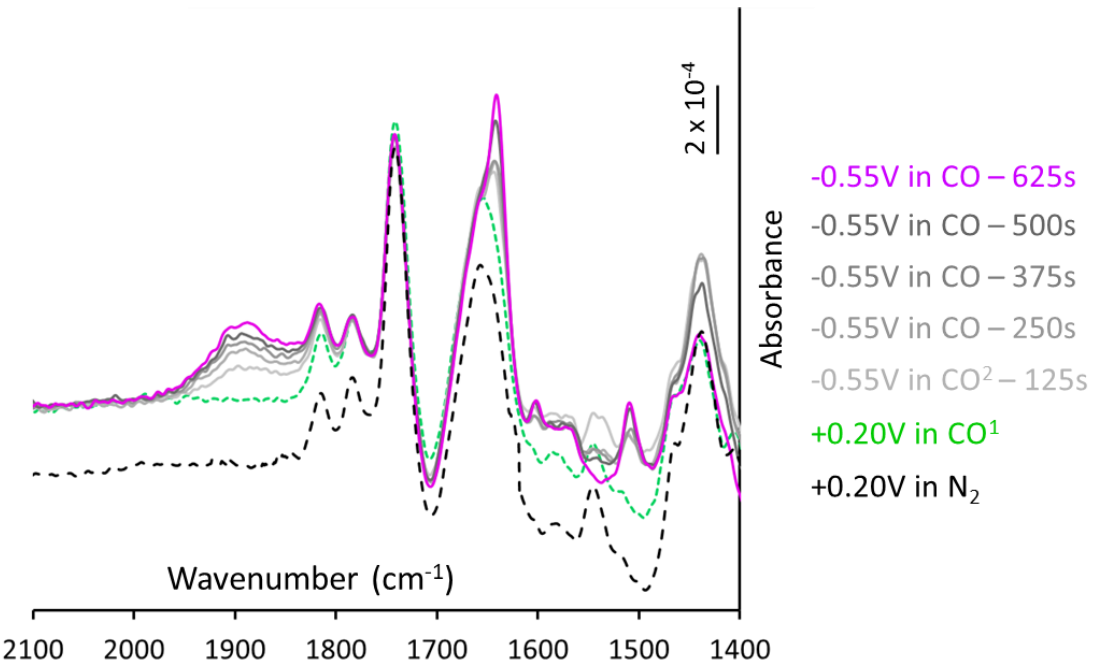

Figure S5. SEIRA spectra of ^{WT}MoFe immobilized on NBD-modified nano-structured Au electrode measured at different potentials in 100 mM MOPS **pD 7** buffers containing 150 µM of MV mediator, in N_2 or CO environment (as stated in the figure legend). Note: when in the CO environment condition 1 was done before 2. *The time mentioned in the legends corresponds to the time of starting the bulk electrolysis.*

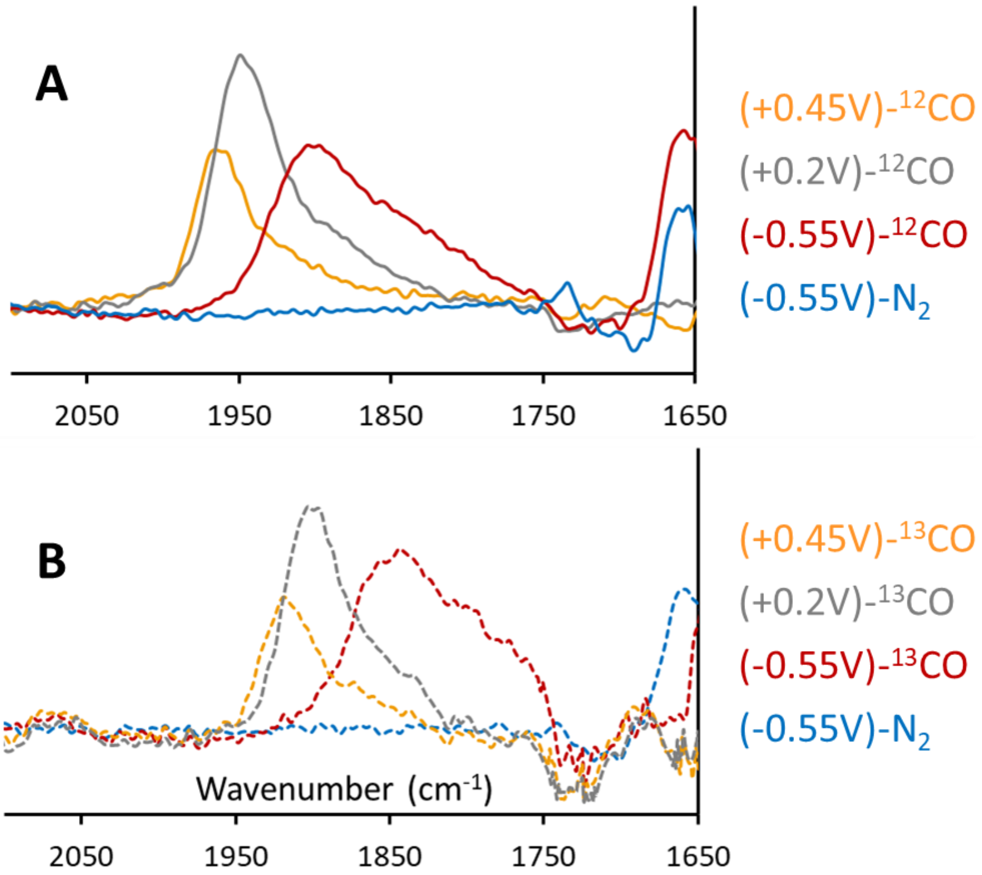

Figure S6. Difference spectra of the SEIRA spectra of ^{WT}MoFe immobilized on NBD-modified nano-structured Au electrode collected at different potentials as measured under ^{12}CO (solid lines) and ^{13}CO (dashed lines) from the corresponding SEIRA spectra measured under OCP in N_2. The difference spectra of the spectra collected at -0.55 V under N_2 from the spectra at OCP is shown as a reference in both the cases.

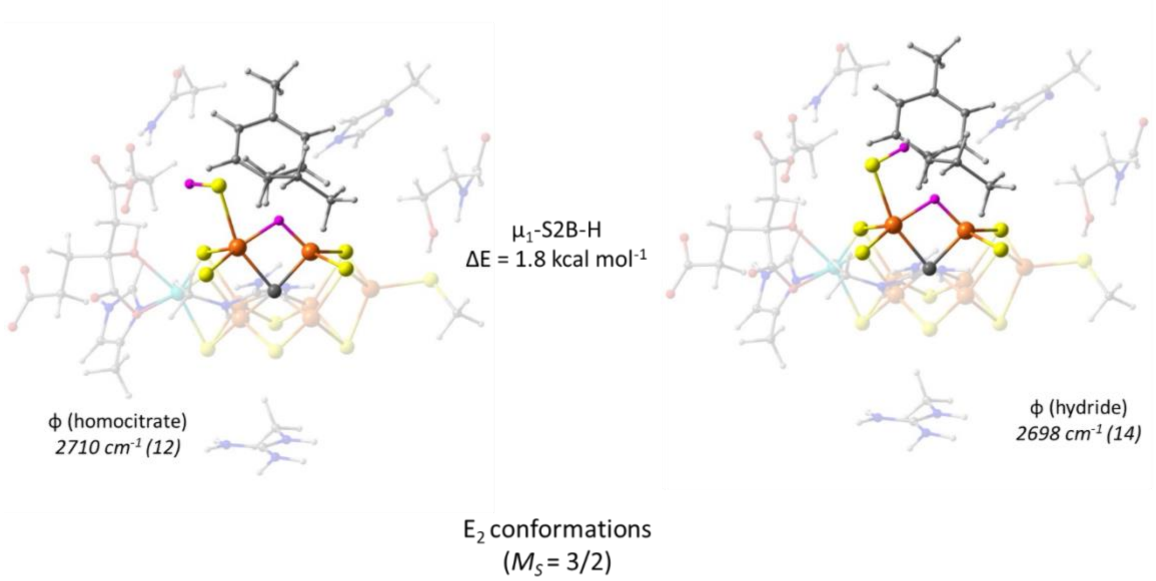

Figure S7. The two possible E_2 conformations calculated (and the torsional barrier between them) obtained for the proposed mechanism for the addition of e^-/H^+ to the FeMoco based on QM/MM calculations. [1] The calculated SH vibrational frequencies are mentioned in the figure along with the stretching intensity (km mol^{-1}) in parenthesis. The atoms of the FeMoco are colored as following; Fe= orange, S= yellow, C= grey and H= magenta.

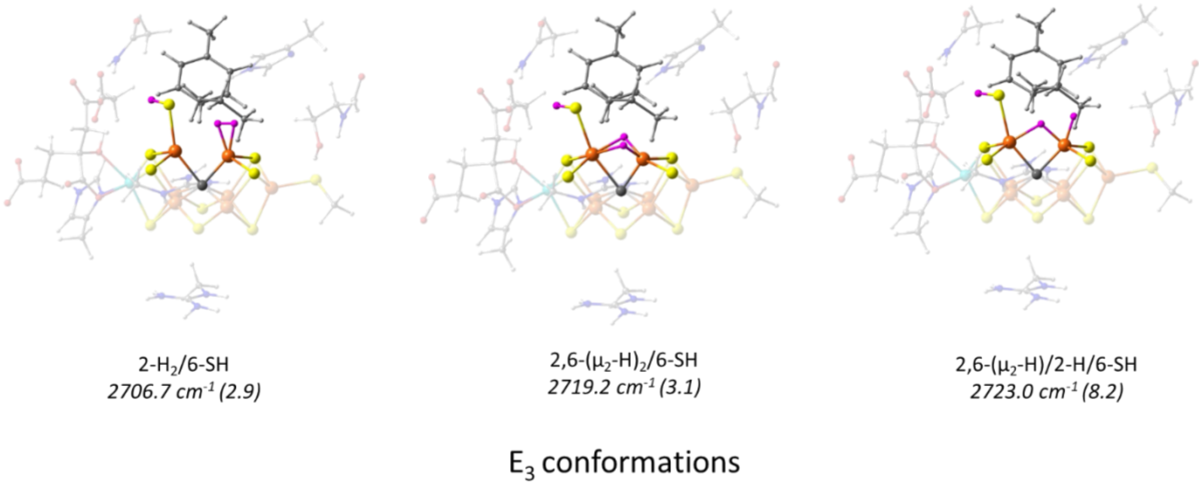

Figure S*8*. The possible E_3 isomers obtained for the proposed mechanism for the addition of e^-/H^+ to the FeMoco based on QM/MM calculations.[2] The calculated vibrational frequencies are mentioned in the figure along with the stretching intensity (km mol^{-1}) in parenthesis. The atoms of the FeMoco are colored as following; Fe= orange, S= yellow, C= grey and H= magenta.

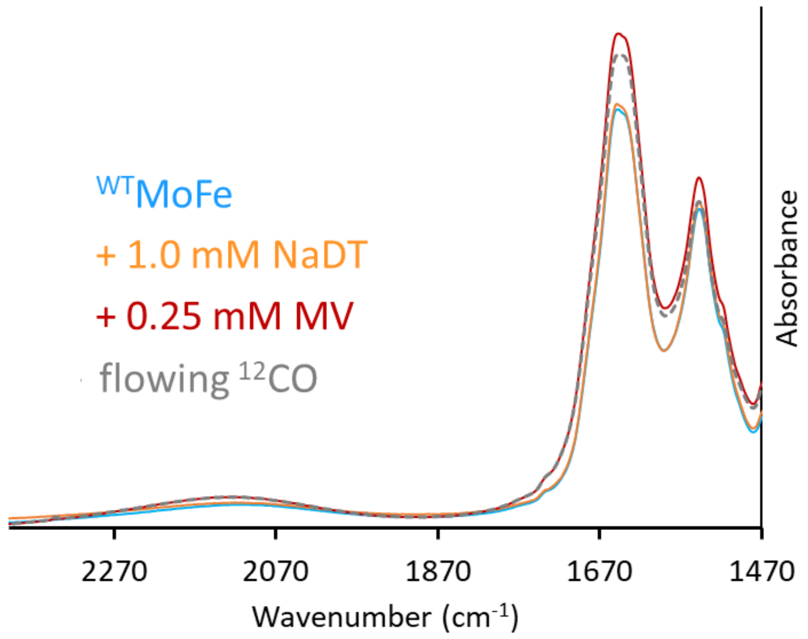

Figure S7. ATR-FTIR spectra of ^{WT}MoFe (blue), followed by the addition of NaDT (orange), methyl viologen (MV, red), and CO (grey).

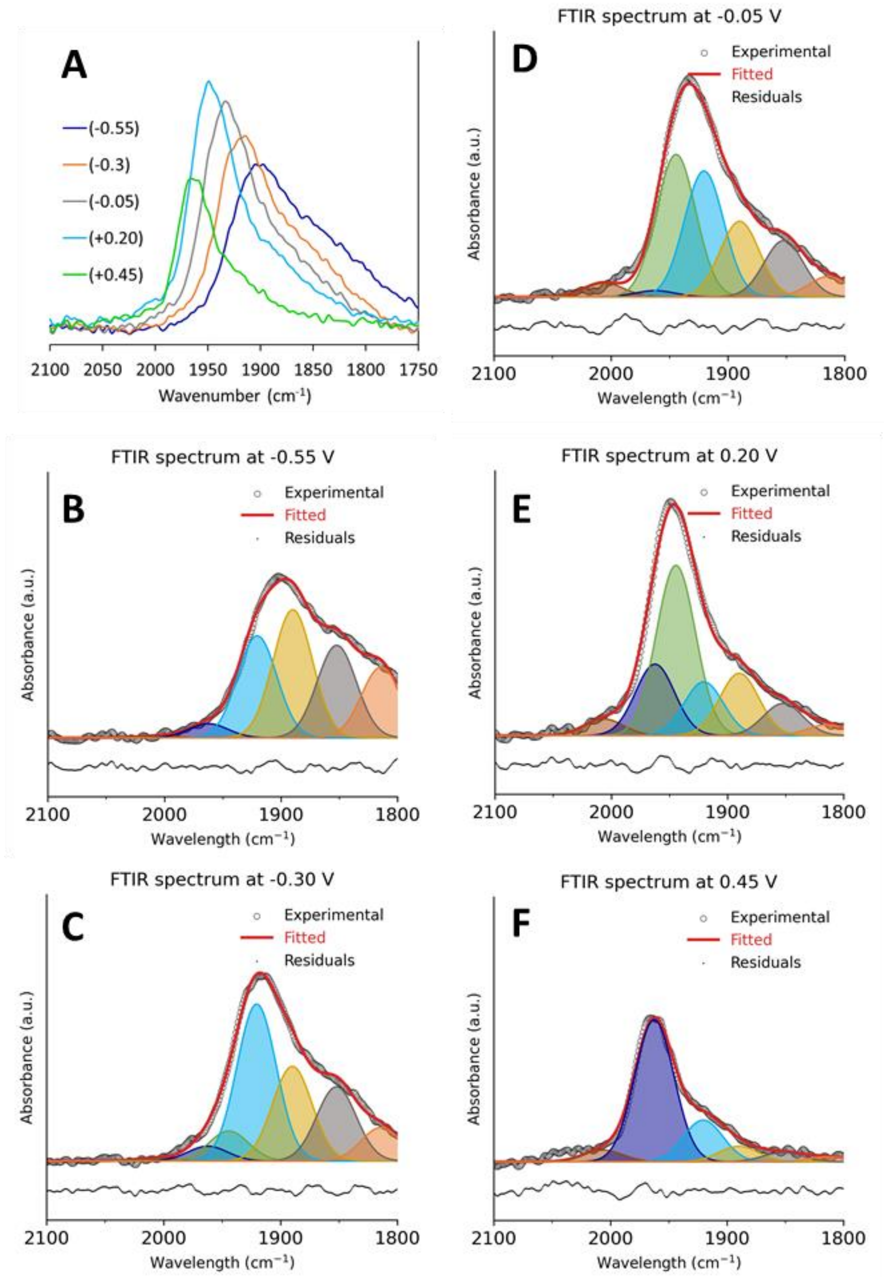

Figure S8. (A) Difference spectra of the SEIRA spectra (as in figure 7A) of ^{WT}MoFe immobilized on NBD-modified nano-structured Au electrode collected at different potentials under ^{12}CO environment in 100 mM MOPS pH 7 buffers containing 150 µM of MV mediator from the corresponding SEIRA spectra measured under OCP in N_2. (B-F) Global fitting of the difference spectra at different potentials to deconvolute components.

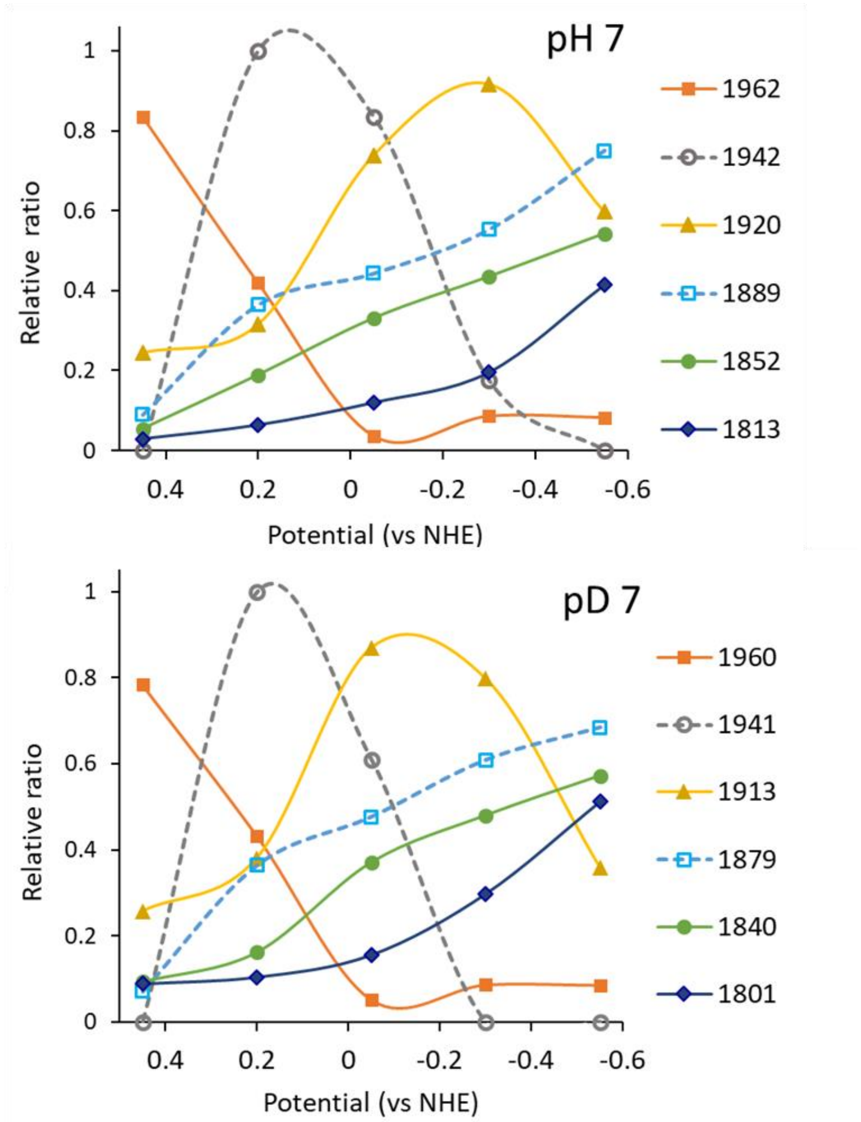

Figure S9. Global fitting results of the SEIRA spectra obtained from the measurements at different potentials under ^{12}CO in pH 7 (top) and pD 7 (bottom) buffer conditions. The lines connecting the points are added for clarity and do not represent fits.

Table S1. The component frequencies (in cm^{-1}) obtained from the global fitting of the CO stretches from different potentials. Gray boxes show the frequencies that shift under pD conditions	
**pH 7**	**pD 7**
1962	1960
1942	1941
1920	1913
1889	1879
1852	1840
1813	1801

References

(1) Thorhallsson, A. Th.; Bjornsson, R. The E2 State of FeMoco: Hydride Formation versus Fe Reduction and a Mechanism for H2 Evolution. *Chem. – Eur. J.* **2021**, *27* (67), 16788–16800. https://doi.org/10.1002/chem.202102730.

(2) Pang, Y.; Bjornsson, R. The E3 State of FeMoco: One Hydride, Two Hydrides or Dihydrogen? *Phys. Chem. Chem. Phys.* **2023**, *25* (31), 21020–21036. https://doi.org/10.1039/D3CP01106B.
